# FBXW7 E3 ligase prevents centriole overduplication by degrading the Plk4 phosphorylated STIL-SAS6 cartwheel assembly

**DOI:** 10.1016/j.jbc.2025.111104

**Published:** 2025-12-24

**Authors:** Ushma Anand, Amit Bloomberg, Pradip Bhattacharjee, Swarnendu Mukhopadhyay, Binshad Badarudeen, Shivani Ramakrishnan, Uri Ben-David, Tapas K. Manna

**Affiliations:** 1 School of Biology, Indian Institute of Science Education and Research, Thiruvananthapuram, Kerala, India; 2 Department of Human Molecular Genetics and Biochemistry, Faculty of Medicine, Tel Aviv University, Tel Aviv, Israel

**Keywords:** centriole, centrosome, FBXW7, STIL, SAS6, aneuploidy

## Abstract

Uncontrolled centriole duplication leads to centrosome amplification and chromosomal instability, but its underlying mechanism is poorly understood. A new centriole is duplicated from a cartwheel-like structure assembled by Plk4-phosphorylated SCL/TAL1-interrupting locus (STIL) and its associated SAS6. Here, we show that depletion of SCF E3 ubiquitin ligase, FBXW7 induces prematured duplication of centrioles *via* excessive stabilization of STIL-SAS6 axis. FBXW7 mediates degradation of STIL-SAS6 axis, and Plk4 kinase activity is required for this degradation. Interestingly, phosphorylation of key Plk4-targeting sites in STIL that drives new centriole assembly by facilitating STIL-SAS6 interaction also stabilizes FBXW7 binding to STIL and promotes degradation of the STIL–SAS6 complex, thus revealing an opposing molecular mechanism to inhibit centriole overduplication. Genomic analyses of cancer cell line data reveal a negative correlation between FBXW7 expression and aneuploidy, as well as a positive correlation between FBXW7 and STIL expression at the mRNA level. Our results thus contribute to improved understanding of the molecular basis of centrosome amplification and aneuploidy.

Centrosome nucleates and organizes microtubules in most animal cells and are composed of two perpendicularly arranged centrioles embedded in a cloud of proteins called pericentriolar material. Centrioles are nine-fold symmetric microtubule-based structures that are duplicated once during the cell cycle to generate two centrosomes that form bipolar mitotic spindles to segregate the chromosomes faithfully (1, 2, 3, 4). Centrosome amplification, caused by overproduction of its centrioles, contributes to aneuploidy in cells by improperly clustering the amplified centrosomes/centrioles and driving cell division with abnormally segregated chromosomes (5). Cells with amplified centrosomes have been shown to develop spontaneous tumors in mice model (6, 7). Structure-function aberrations of centrioles are also linked to diseases such as primary microcephaly and ciliopathies (8, 9, 10, 11).

Centriole duplication begins at G1/S entry of the cell cycle at the proximal side of each of the parental centrioles by forming a striking nine-fold symmetric central cartwheel-like structure. The cartwheel serves as a framework for assembling the early centriolar structure called procentriole with nucleated nine sets of triplet microtubules, which further elongate giving rise to the matured centriole. Studies in multiple organisms demonstrated key role of a conserved protein module consisting of polo-like kinase −4 (Plk4/Sak/ZYG-1), STIL (SCL/TAL1-interrupting locus)/Ana2/SAS5, and spindle assembly abnormal 6 (HsSAS6/D-SAS6/SAS6) in formation of the cartwheel. During G1 stage, Plk4 encircles the mother centriole base as a diffused ring and afterward, and during G1 to S transition, the ring transforms into a concentrated dot from where the procentriole formation is initiated (12, 13, 14, 15, 16). Plk4 kinase activation is critical for its “ring to dot” transition, and it is facilitated by Plk4 binding to STIL (17, 18). Plk4-mediated phosphorylation in the conserved STAN domain of STIL promotes its binding to SAS6, whose nine homodimers oligomerize to form the cartwheel (18, 19, 20, 21, 22, 23). Increasing evidences suggest that Plk4 activates corecruitment of STIL and SAS6 in the form of complex upon phosphorylation in the SAS6-interacting conserved STAN domain of STIL (24, 25, 26, 27). Assembly of the template cartwheel structure by recruiting STIL–SAS6 complex, therefore, is a critical step for new centriole generation. Elevated expression levels and centrosomal abundance both STIL and SAS6 have been shown to drive unscheduled formation of the extra cartwheels leading to supernumerary centrioles (25, 28). Both proteins exhibit aberrant expressions and mutations in cancers and developmental diseases (29, 30, 31). Notably, STIL mutations are also linked with primary microcephaly caused by supernumerary centrioles (32). Mechanism of how STIL–SAS6 complex at an optimal threshold level is maintained in order to license just one, but not more, procentriole formation from the parent centriole is less defined.

Evolutionarily conserved E3 ubiquitin ligase of SCF (SKP1-Cul-1-F-box protein) family FBXW7 localizes to centrioles, and its loss of function leads to overproduction of centrioles/centrosomes in human cells (33, 34, 35). FBXW7 is mutated in numerous cancers, and its deletion induces chromosomal instability (36, 37, 38, 39, 40). Here, we show that FBXW7 controls centriole amplification by regulating stability of the STIL-SAS6 axis by inducing proteasomal degradation of both the proteins in their associated form in human cells. FBXW7 depletion induces stabilization of STIL and SAS6 in the form of multiple foci at the centrosome leading to overduplicated centrioles. FBXW7 interacts with STIL through SAS6-binding conserved STAN domain of STIL, whose deletion renders both STIL and SAS6 resistant toward FBXW7-mediated degradation. Plk4 kinase activity is essential for FBXW7-mediated STIL-SAS6 degradation. Furthermore, phosphorylation at the Plk4-targeting sites in a FBXW7 consensus phosphodegron motif in STIL STAN domain that promotes STIL-SAS6 binding and new centriole assembly plays a critical role in STIL-SAS6 degradation and centriole duplication control. The results demonstrate that FBXW7 regulates centriole/centrosome copy number *via* targeted destruction of the centriole assembly-promoting phosphorylated STIL and its associated SAS6.

## Results

### FBXW7 regulates STIL stability to control aberrant centriole number

**Figure 1:**
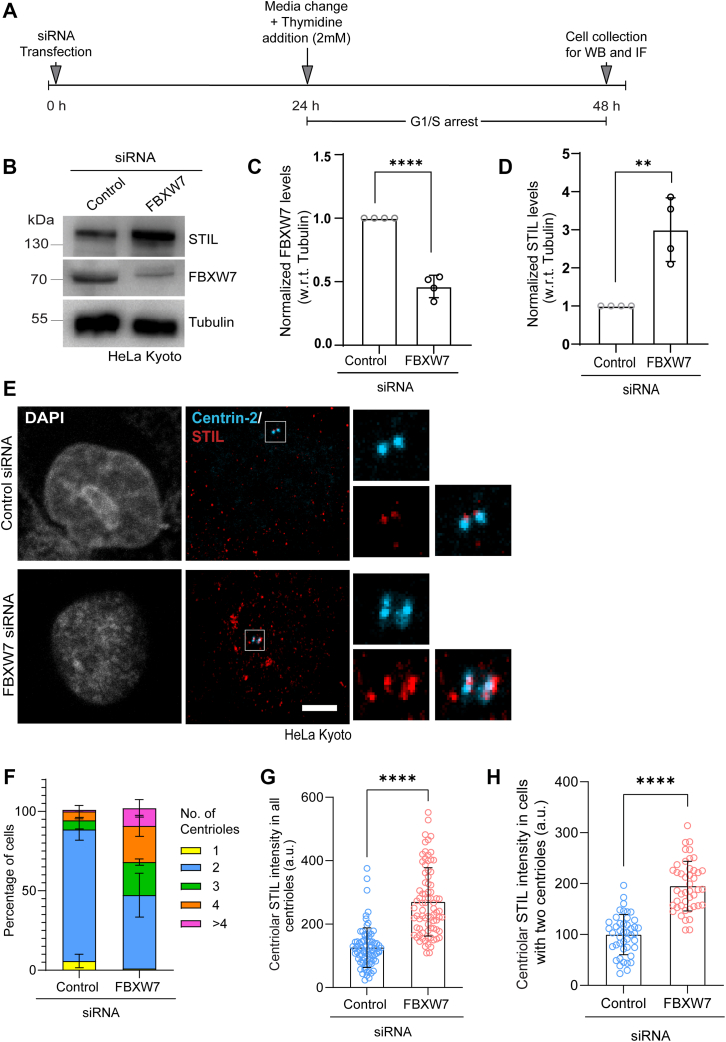
**FBXW7 depletion induces centriole amplification by stabilizing STIL at the centrosome.***A*, schematics of STIL siRNA transfection followed by single thymidine treatment and collection of G1/S synchronized cells for Western blot (WB) and immunofluorescence (IF) microscopy. *B*, lysates of HeLa Kyoto cells after transfection with control or FBXW7 siRNA followed by G1/S synchronization were probed for STIL and FBXW7 by Western blot. Tubulin was probed as control. *C*, plot shows the levels of FBXW7 normalized to tubulin in control *versus* FBXW7 siRNA-treated HeLa Kyoto cells. ∗∗∗∗*p* < 0.0001(Unpaired parametric *t* test). Data = mean ± S.D. *D*, levels of STIL normalized to tubulin as of b were plotted. N = 4, ∗∗*p* = 0.0030 (Unpaired parametric *t* test), data = mean ± S.D. *E*, representative confocal images of G1/S synchronized HeLa Kyoto cells depleted of FBXW7 by siRNA showing STIL localization as multiple foci as supernumerary centrioles (>4). Centrin-2 was stained as a centriole marker. Scale bar = 5 μm. *F,* plot of % cells with different centriole number in control *versus* FBXW7-depleted HeLa Kyoto cells. n = ∼75 cells in each, N = 3. *G*, plots of centrosome-localized STIL intensity in control *versus* FBXW7-depleted G1/S synchronized HeLa Kyoto cells for the conditions as of E. Data = mean ± S.D. n = ∼75 cells in each, N = 3, ∗∗∗∗*p* < 0.0001(Unpaired parametric *t* test). *H*, plots of centrosomal STIL intensity of control *versus* FBXW7-depleted cells containing two centrioles. Data = mean ± S.D., n = ∼45 cells, N = 3, ∗∗∗∗*p* < 0.0001(Unpaired parametric *t* test). STIL, SCL/TAL1-interrupting locus.

We assessed how FBXW7 depletion could regulate STIL and SAS6 in human cells. HeLa Kyoto cells were depleted of FBXW7 by siRNA, and the cellular levels of both the proteins were analyzed from the lysates of cells synchronized at G1/S by thymidine treatment (2 mM) (Fig. 1*A*). There was a marked increase (∼3 ± 0.4 folds) of STIL level in the FBXW7-depleted cells as compared to control siRNA-treated cells (Fig. 1, *B* and *D*). The level of FBXW7 depletion in these cells was about ∼55 ± 0.1% (Fig. 1*C*). Specificity of FBXW7 depletion-induced effect was further supported by rescue experiment, in which exogenously expressed siRNA-resistant Flag-FBXW7 in FBXW7 siRNA-treated HeLa Kyoto cells could repress STIL level to the level as control (Fig. S1*A*). Similar STIL stabilization was observed in FBXW7-depleted HEK293T cells (Fig. S1*B*). Similar to STIL stabilization, FBXW7 depletion also led to increased stabilization of SAS6 in HEK293T cells (Fig. S1*C*). Under similar condition, Plk4 level did not change as compared to control HEK293 cells (Fig. S1*C*). We also verified STIL level by depleting FBXW7 using SMART pool siRNA and found similar stabilization of STIL (Fig. S1*D*). We then examined how the suppression of FBXW7 affects centriolar localization of these proteins. Analysis of G1/S synchronized FBXW7-depleted HeLa Kyoto cells showed enhanced STIL localization at the centrosome/centrioles (Figs. 1*E*, S1*E*) with concomitant increase of centriole number in those cells. Percentage of cells with more than two centrioles (three and above) with each having STIL foci localized was increased from ∼12% in control to ∼55% in FBXW7-depleted condition (Fig. 1*F*). Similar centriole amplification (centriole number 3 and more increased from ∼5% to ∼40%) defect with aggravated STIL was apparent in U2OS cells in response to FBXW7 depletion (Fig. S1, *F* and *G*). Intensity analysis showed ∼ 45% increased centrosomal STIL level in FBXW7-depleted cells as compared to control HeLa Kyoto cells (Fig. 1*G*). Even comparison of the same in cells possessing exclusively two centrioles in control *versus* FBXW7-depleted cells also showed a similar trend of increased STIL supporting that STIL is overstabilized at the individual centriole level (Fig. 1*H*). Under similar condition, multiple SAS6 foci with visibly increased intensity in each was also evident in HeLa Kyoto cells in response to FBXW7 depletion (Fig. S1*H*). In an earlier study, overexpression of another FBXW family ubiquitin ligase, βTrCP was shown to reduce STIL level in interphase cells, but this study also reported that depletion of βTrCP did not cause overaccumulation of STIL (41). The authors speculated possible roles of other ubiquitin ligases. We also verified the effect of βTrCP depletion in comparison with that of FBXW7 depletion under similar condition in the G1/S synchronized HeLa Kyoto cells. In corroboration with the earlier report, siRNA-mediated βTrCP depletion indeed did not result in significant stabilization of STIL, though FBXW7 depletion exhibited a robust effect on the same (Fig. S1*I*).

### FBXW7 depletion results in multiple premature centrioles

**Figure 2:**
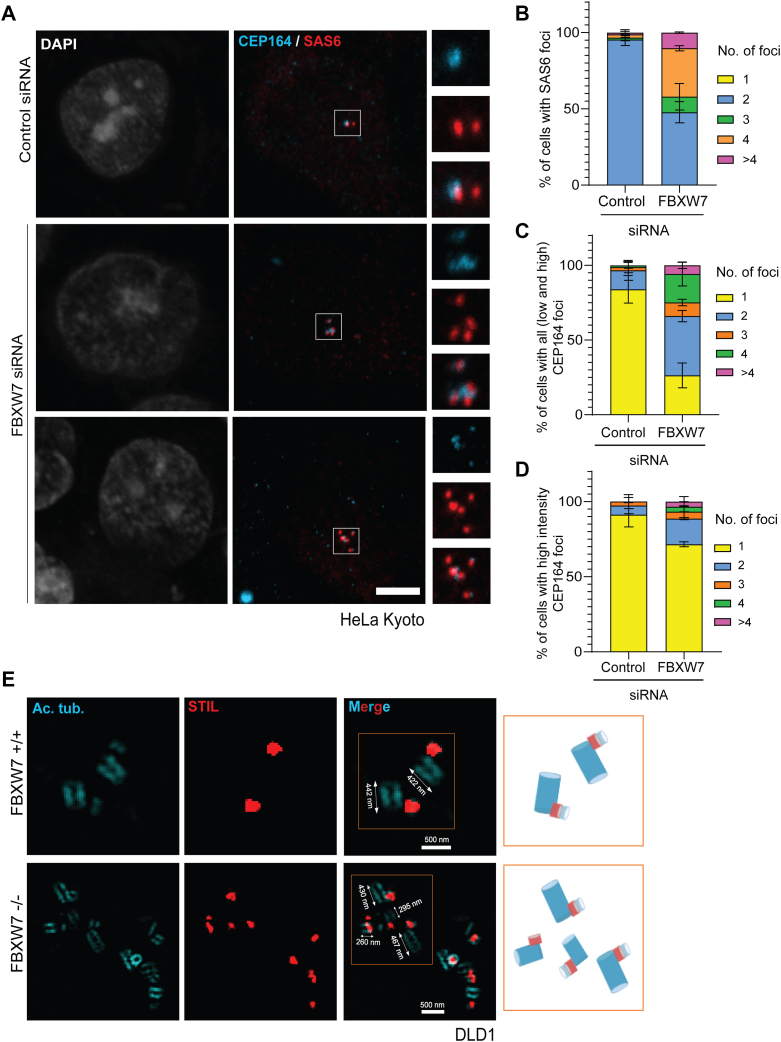
**FBXW7 depletion induces supernumerary centrioles with multiple mother-daughter pairs.***A,* representative confocal microscopic images of G1/S synchronized HeLa Kyoto cells treated with control or FBXW7 siRNA (48 h) stained for CEP164 and SAS6. Scale bar = 5 μm. While the vast majority (>∼95%) of control cells carry two centrioles, ∼ 50% FBXW7-depleted cells possess supernumerary (3 and more) centrioles with concomitant reduction of cells with two centrioles as shown. *B*, plot of percentage of cells with different numbers of SAS6 foci as indicated in control *versus* FBXW7 siRNA-treated G1/S synchronized HeLa Kyoto cells. *C*, plot of percentage of cells with different numbers of CEP164 foci (low and high intensity) as specified in control *versus* FBXW7-depleted G1/S synchronized HeLa Kyoto cells. *D*, plot of percentage of cells with different numbers of high intense (∼1.5 folds more than the low intense foci) CEP164 foci as specified in control *versus* FBXW7-depleted G1/S synchronized HeLa Kyoto cells. n = ∼90 cells, N = 3. *E*, FBXW7 ^+/+^ (WT) and FBXW7 ^−/−^ knockout (KO) DLD-1 cells were processed for expansion microscopy imaging (Experimental Procedures). The images of an early S and G2 stage-specific FBXW7 WT and FBXW7 KO cells, respectively, are shown. Lengths of the parental centrioles are also shown. Note the procentriole formation from some of the parental centrioles in FBXW7 KO cells that are smaller (premature stage) than the usual length. Scale bar = 500 nm.

To gain insights into how the extra centrioles are generated in FBXW7-depleted cells, G1/S synchronized control and FBXW7 siRNA-treated HeLa Kyoto cells were stained for mother centriole appendage protein, CEP164, together with SAS6. As anticipated, the control cells showed a single CEP164 and two SAS6 foci, indicating the presence of a single mother out of the two centrioles. However, in FBXW7-depleted cells with three or more centrioles, multiple SAS6 positive foci were evident with several of them positive with CEP164 staining (Figs. 2*A*, S1*J*). While the percentage of cells carrying a single CEP164 foci was decreased from 80% in control to ∼ 20% in the FBXW7-depleted cells, the same with two or more than two CEP164 foci (2, 3, 4, and > 4) was increased from ∼ 20% from control to nearly 80% in the FBXW7-depleted cells (Fig. 2*C*). Similar trend was observed in case of SAS6 localization as the percentage of cells with three or more SAS6 positive foci were increased from less than 2% in control to nearly 45% in the FBXW7-depleted condition (Fig. 2*B*). Interestingly, though many of the extra centrioles were positive for CEP164 in FBXW7-depleted cells, only a single or two CEP164 foci appeared to be of high intensity (∼1.5 folds higher intensity than the lower intensity foci), and the others appeared as less intense foci (Fig. 2, *A* and *D*). The additional less intense CEP164-positive foci are indicative of incompletely matured mother centrioles. This is also suggestive of early duplication of the centrioles prior to their complete maturation. To further confirm such aggravated centriole duplication cycle, we imaged the centrioles by staining with acetylated-tubulin in FBXW7 WT (+/+) *versus* FBXW7 −/− knockout (KO) DLD-1 cells by expansion microscopy. While the FBXW7 WT cells showed two mother centriole-procentriole pairs, the FBXW7 KO cells were seen to possess multiple (>2) mother centriole-procentriole pairs with each of the procentrioles stained positive with STIL, strongly supporting reduplication of centrioles in multiple rounds (Fig. 2*E*). Some among the extra-generated mother centrioles in the FBXW7 KO cells appeared to be smaller (∼30–40%) in length than those of the FBXW7 WT cells or the initial parental pairs in the same cells, yet they appeared to make the procentrioles from their proximal ends, substantiating the possibility that procentriole assembly occurs prior to complete maturation of the parent centrioles in the FBXW7-deleted cells.

### FBXW7 overexpression leads to reduction of STIL level

**Figure 3:**
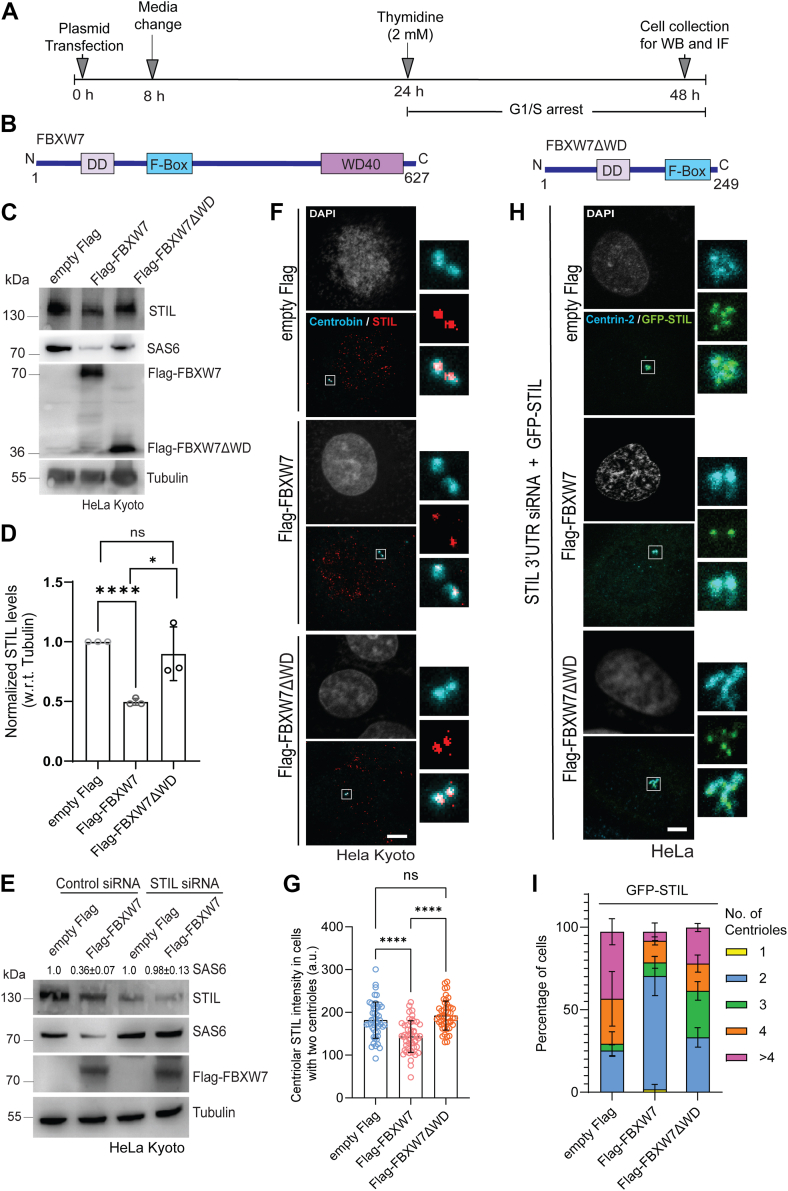
**FBXW7 suppresses supernumerary centrioles induced by exogenously expressed STIL.***A*, design of the experiment of Flag-FBXW7 or control empty Flag plasmid transfection followed by G1/S synchronization by thymidine is shown. *B*, schematic representations of Flag-FBXW7 and Flag-FBXW7ΔWD. Dimerization domain (DD), F-box, and WD40 repeat domains are shown. *C*, lysates of HeLa Kyoto cells transfected with Flag-FBXW7 or Flag-FBXW7ΔWD were subjected to Western blot under G1/S synchronized condition to assess STIL levels. SAS6, Flag-tagged FBXW7 proteins, and tubulin (marker) were also probed. *D*, plot of STIL levels normalized with respect to tubulin in HeLa Kyoto cell lysates as shown in C. N = 3, ∗∗∗∗*p* < 0.0001, ∗*p* < 0.05 (Unpaired parametric *t* test). *E*, lysates of control siRNA *versus* siRNA-mediated STIL-depleted HeLa Kyoto cells transfected with Flag-FBXW7 or empty Flag plasmid and synchronized at G1/S were probed for the levels of SAS6 and STIL. SAS6 intensity values normalized with respect to marker tubulin are shown. N = 3. *F,* representative confocal microscopy images of HeLa Kyoto cells expressed with empty Flag or Flag-FBXW7 or Flag-FBXW7ΔWD were stained for STIL and centrobin under G1/S synchronized condition. DNA was stained with DAPI. Images of cells with centrioles (two centrobin foci) are shown to visualize the differences of centriole-localized STIL intensity. *G*, intensity plot of centriolar STIL in cells with two centrioles under expression of control Flag or Flag-FBXW7 proteins as specified. Data = mean ± S.D., n = ∼50 cells in each, *p* < 0.0001(Unpaired parametric *t* test). *H,* representative confocal images of HeLa cells overexpressed with GFP-STIL under depletion of endogenous STIL by STIL 3′UTR siRNA were expressed with control Flag or Flag-FBXW7 or Flag-FBXW7ΔWD and stained with GFP and Centrin-2 antibody. GFP-STIL overexpression-induced supernumerary centrioles (>2) defect was suppressed upon Flag-FBXW7 overexpression. *I*, plot of percentage of cells with different number of centrioles in GFP-STIL-overexpressed cells under control Flag or Flag-FBXW7 or Flag-FBXW7ΔWD expression as shown in *H*. Data = mean ± S.D. N = 3, n = ∼ 70 cells in each. Scale bar for all images = 5 μm. STIL, SCL/TAL1-interrupting locus.

Next, we assessed the effect of FBXW7 overexpression on the stability of STIL. Lysates of G1/S synchronized HeLa Kyoto cells and HEK293T cells after 48 h of Flag-tagged FBXW7 (Fig. 3, *A* and *B*) or control Flag transfection were analyzed for the levels of endogenous STIL. STIL level was markedly reduced in cells expressed with Flag-FBXW7 as compared to that of control (Fig. 3*C*), and –∼ 45 ± 0.2% reduction of STIL was observed (Fig. 3*D*). Level of SAS6 also showed a similarly marked reduction upon exogenous Flag-FBXW7 expression (Fig. 3*E*). Overexpression of a FBXW7 deletion mutant that is devoid of the substrate-binding WD repeat domain (42), Flag-FBXW7-ΔWD, failed to exert such an effect on STIL and SAS6 levels (Fig. 3, *C* and *D*), suggesting critical role of the WD domain. Interestingly, Flag-FBXW7 overexpression failed to induce SAS6 degradation in the STIL-depleted G1/S synchronized HeLa Kyoto cells, suggesting that FBXW7 likely targets the STIL-associated form of SAS6 (Fig. 3*E*). Next, we examined the effect of exogenous Flag-FBXW7 expression on the level of STIL at the centrosome/centrioles. HeLa Kyoto cells were transfected with Flag-FBXW7 or empty Flag for 48 h, and the cells after synchronization at G1/S by thymidine were imaged for centriolar/centrosomal STIL level. Centrobin was stained as a marker. Centrosomal STIL level was significantly reduced in the Flag-FBXW7-expressed cells as compared to control (Figs. 3, *F* and *G*, Fig. S2*A*). Similar reduction was also apparent for centriolar SAS6 (Fig. S2, *B* and *C*). Expression of Flag-FBXW7-ΔWD did not exert such an effect on STIL at the centrioles/centrosome again substantiating the role of the substrate-binding domain (Figs. 3, *F* and *G*, Fig. S2*A*). Next, we examined how the elevated FBXW7 level exerts effects on centriole amplification induced by increased STIL expression. This was accomplished by exogenously expressing GFP-STIL in cells with simultaneous depletion of endogenous STIL by siRNA. HeLa cells after 8 h of transfection with STIL 3′ UTR siRNA were cotransfected with GFP-STIL and Flag-FBXW7 or Flag-FBXW7-ΔWD for another 48 h prior to image the cells for centriolar GFP-STIL and Centrin-2 in G1/S synchronized condition. In majority of the GFP-STIL overexpressed cells transfected with empty Flag plasmid or Flag-FBXW7-ΔWD, overamplified (3 and more) centrioles were apparent in ∼70 to 75% cells (Figs. 3, *H* and *I*, Fig. S2*D*). The same was reduced markedly (to ∼ 30%) in the cells expressed with Flag-FBXW7 (Fig. 3, *H* and *I*), indicating that centriole amplification induced by the exogenously expressed GFP-STIL was suppressed by Flag-FBXW7.

### FBXW7 interacts with and ubiquitinates STIL

**Figure 4:**
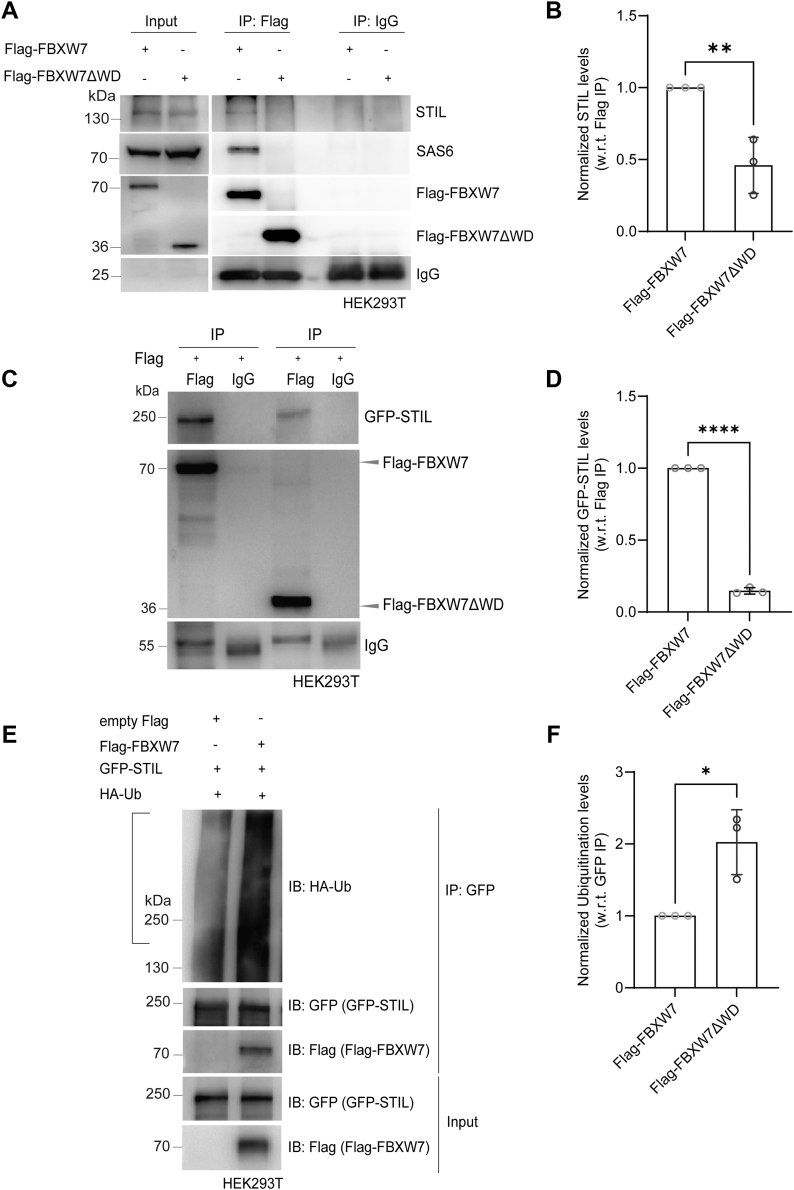
**FBXW7 interacts with and targets STIL for ubiquitin-mediated degradation.***A*, lysates of G1/S synchronized HEK293T cells expressed with Flag-FBXW7 or Flag-FBXW7ΔWD for 48 h and after treatment with MG132 (25 μM) during last 6 h were subjected to Flag pulldown and the samples were probed for STIL, SAS6, and the Flag-tagged proteins. *B*, levels of STIL association with the Flag-tagged FBXW7 proteins in Flag-FBXW7 or Flag-FBXW7ΔWD-expressed HEK293T cells are plotted. N = 3, Data = mean ± S.D., ∗∗*p* = 0.0086 (Unpaired parametric *t* test). *C*, lysates of G1/S synchronized GFP-STIL overexpressed HEK293T cells transfected with Flag-FBXW7 or Flag-FBXW7ΔWD followed by MG-132 (6h) treatment were subjected to Flag pulldown, and the presence of GFP-STIL was probed by Western blot. *D*, levels of GFP-STIL associated with the Flag-tagged FBXW7 proteins in Flag-FBXW7- or Flag-FBXW7ΔWD-expressed HEK293T cells were plotted. Data = mean ± S.D., ∗∗∗∗*p* < 0.0001(Unpaired parametric *t* test), N = 3. *E*, GFP-STIL- expressed HEK293T cells transfected with HA-Ub together with either Flag-FBXW7 or empty Flag plasmid for 12 h followed by G1/S synchronization by thymidine (18h), and then treatment with MG-132 (6h) were subjected to immunoprecipitation by GFP-trap beads. Immunoprecipitated GFP-STIL in Flag-FBXW7 or empty Flag transfected cells was probed in a 8% SDS-PAGE gel to stain GFP-STIL using GFP antibody and in a 6% gel for ubiquitinated proteins by using HA antibody. *F*, levels of ubiquitinated proteins based on HA-Ub intensity in the selected region as shown in empty Flag and Flag-FBXW7-expressed condition as of panel E were plotted. Data = mean ± S.D. N = 3, ∗*p* < 0.05 (unpaired parametric *t* test). STIL, SCL/TAL1-interrupting locus; HA-Ub, HA-tagged ubiquitin.

We next sought to investigate FBXW7 interaction with STIL. Lysates of G1/S synchronized HeLa Kyoto cells after transfection with Flag-FBXW7 or control Flag were subjected to Flag pulldown and then analyzed for the presence of STIL. Flag pulldown showed association of Flag-FBXW7 with endogenous STIL (Fig. 4*A*). Flag pulldown from the Flag-FBXW7ΔWD-expressed cells showed no detectable association of FBXW7ΔWD with STIL (Fig. 4, *A* and *B*). Under the same condition, SAS6 was also found to be associated with Flag-FBXW7 but not with Flag-FBXW7ΔWD (Fig. 4*A*). Next, we checked if FBXW7 interacts with the exogenously expressed GFP-STIL in cells. Lysates of G1/S synchronized HEK293T cells overexpressed with GFP-STIL followed by Flag-FBXW7 transfection were subjected to Flag pulldown and probed for the presence of GFP-STIL. Flag-FBXW7 pulldown showed strong presence of GFP-STIL (Fig. 4*C*). Moreover, Flag pull-down from the lysates of cells expressed with Flag-FBXW7ΔWD showed much reduced association of GFP-STIL with Flag-FBXW7ΔWD (Fig. 4*D*). To verify if FBXW7-mediated suppression of STIL level is due to ubiquitin-mediated degradation of STIL, ubiquitination of STIL was assessed in control *versus* Flag-FBXW7 overexpressed HEK293T cells. GFP-STIL-expressed HEK293T cells were transfected with Flag-FBXW7 or empty Flag plasmid along with HA-tagged ubiquitin (HA-Ub) for 48 h, and then the level of GFP-STIL ubiquitination was assessed based on HA-Ub staining in the GFP-STIL immunoprecipitates from the G1/S synchronized cell lysate added with proteasome inhibitor, MG132 (25 μM). HA-Ub level in the GFP-STIL immunoprecipitate from the Flag-FBXW7 overexpressed cells appeared substantially higher as compared to that of control empty Flag-expressed cells (Fig. 4, *E* and *F*). To visualize the ubiquitinated protein bands, samples of equal volume of the IP samples were run separately in a low percentage gel (Experimental Procedure), and the ubiquitinated proteins were probed. Image of the whole immunoblot of the HA-Ub stained proteins is shown in Figure S2*E*.

### FBXW7 mediates degradation of STIL through its STAN domain

**Figure 5:**
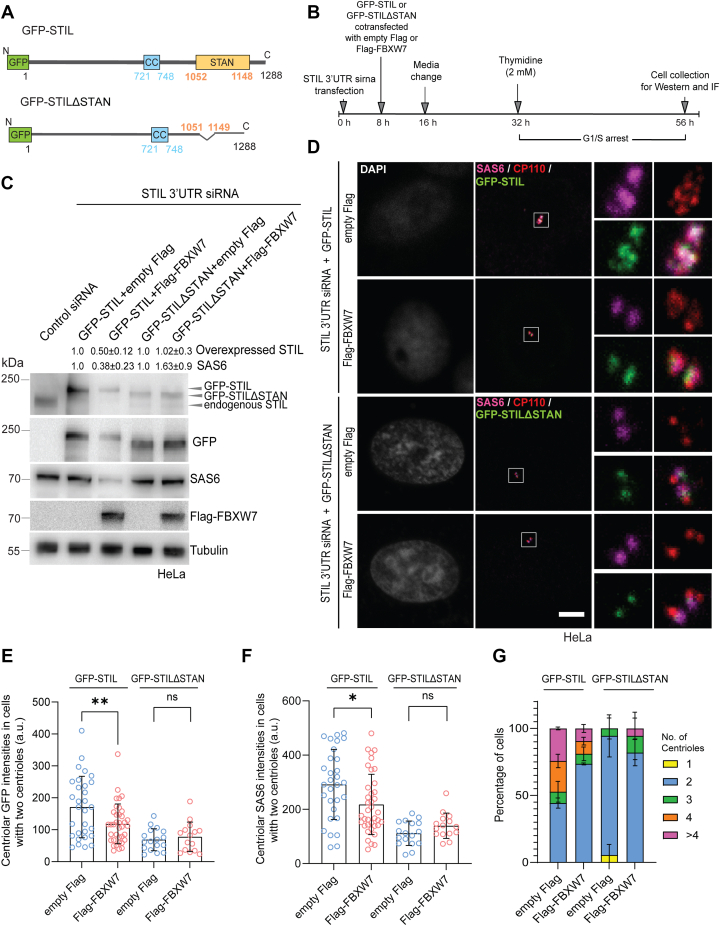
**STIL degradation by FBXW7 is mediated *via* STAN domain of STIL.***A*, schematic representation of GFP-tagged human WT STIL (GFP-STIL) and its STAN domain-deleted form (GFP-STILΔSTAN). Plk4-binding regions (CC) are also shown. *B*, schematics of transfection and cell synchronization followed for panels *C* and *D* are shown. *C*, lysates of GFP-STIL or GFP-STILΔSTAN-expressed STIL 3′ UTR siRNA-treated HeLa cells cotransfected with Flag-FBXW7 or empty Flag followed by G1/S synchronization by thymidine (as shown in the schematic) were subjected to Western blot. Tubulin was probed as loading control. Quantitation of GFP-STIL or GFP-STILΔSTAN and SAS6 bands normalized to tubulin based on three experiments are shown on top (unpaired parametric *t* test). *D*, representative confocal images of GFP-STIL or GFP-STILΔSTAN-expressed STIL 3′ UTR siRNA-treated HeLa cells transfected with empty Flag or Flag-FBXW7 were synchronized at G1/S and stained for SAS6, CP110 (as marker), and the GFP-STIL proteins. Scale bar = 5 μm. *E*, quantification plots of centrosome-localized GFP-STIL or GFP-STILΔSTAN in cells as shown in d are shown. Cells containing two centrioles were considered for quantification. *F*, quantification plots of centrosome-localized SAS6 in cells as shown in *D* are shown. Data for E and F = mean ± S.D., n = ∼60 cells each for GFP-STIL and n = ∼30 cells each for GFP-STILΔSTAN condition. ∗∗*p* < 0.01 and ∗*p* < 0.05 (Unpaired parametric *t* test). *G,* quantitation plot of cells with centrioles in GFP-STIL or GFP-STILΔSTAN-expressed STIL 3′ UTR siRNA-treated HeLa cells transfected with empty Flag or Flag-FBXW7. Data = mean ± S.D., n = ∼ 40 to 70 cells. STIL, SCL/TAL1-interrupting locus.

Plk4-mediated phosphorylation in the STAN domain (amino acids 1052–1148) of STIL in its C terminus promotes SAS6 recruitment to the centrioles, and SAS6 binding to STIL involves the STAN domain (Fig. 5*A*) (18, 19, 43, 44). We therefore investigated the role of the STAN domain in FBXW7-mediated STIL regulation. Lysates of G1/S synchronized HEK293T cells depleted of endogenous STIL by 3′ UTR siRNA and cotransfected with Flag-FBXW7 and GFP-STIL or GFP-STILΔSTAN, which is devoid the STAN domain, were subjected to Western blot to examine the difference, if any, in the levels of GFP-STILΔSTAN under Flag-FBXW7 expression as compared to the empty Flag-expressed condition (Fig. 5*B*). In order to determine the exclusive effect of overexpression of the exogenously expressed GFP-STIL-WT and the GFP-STIL-ΔSTAN mutant on centriole duplication without interference of the endogenous STIL protein, cells were cotransfected with the GFP-STIL plasmids and the 3′-UTR siRNA of STIL for depleting the endogenous STIL with simultaneous expression of the exogenous STIL proteins. The level of overexpression of the GFP-STIL proteins over the endogenous STIL protein of control cells was in the range of ∼1.5 ± 0.4 (SD) folds (three experiments). Unlike the full-length GFP-STIL, whose level was substantially reduced under Flag-FBXW7 expression, little or no change in the level of GFP-STILΔSTAN in the Flag-FBXW7-expressed condition as compared to empty Flag-expression was observed (Fig. 5*C*), supporting that FBXW7-mediated STIL degradation is mediated *via* the STAN domain. The amount of endogenous STIL depletion and the level of exogenously expressed STIL are shown in Figure 5*C*. It is to note that GFP-STILΔSTAN band intensity appears faint in the empty Flag-expressed condition, when stained with STIL antibody as compared to staining by GFP antibody. It is likely due to weak affinity of STIL antibody as it is specific to the C-terminal region of STIL near to the STAN domain. GFP-STILΔSTAN was also found to be stable against Flag-FBXW7-mediated degradation in cells overexpressed with GFP-STILΔSTAN without depletion of the endogenous STIL (Fig. S3*A*). Interestingly, absence of the STAN domain also attenuated SAS6 degradation by Flag-FBXW7, whereas its level was substantially reduced in the full-length GFP-STIL-transfected cells upon expression of Flag-FBXW7 (Fig. 5*C*). Consistent with regulation of cellular levels of STIL and SAS6, centrosomal localization of GFP-STIL was markedly reduced by Flag-FBXW7 in GFP-STIL overexpressed HeLa cells under endogenous STIL-depletion (Figs. 5, *D* and *E*, S3*B*). GFP-STILΔSTAN though could localize to centrosome, but it was visibly less as compared to the WT GFP-STIL in the control cells. This is expected as the Plk4-mediated phosphorylation in the STAN domain stabilizes STIL at the centrosome and is consistent with earlier study (26). However, centrosomal GFP-STILΔSTAN level was not further reduced upon expression of exogenous Flag-FBXW7 (Fig. 5, *D* and *E*). Interestingly, Flag-FBXW7 expression in GFP-STIL overexpressed cells depleted of endogenous STIL also showed a marked reduction of centrosomal SAS6 localization as compared to empty-Flag expressed GFP-STIL cells, but it failed to exert so in the GFP-STILΔSTAN-overexpressed cells (Fig. 5, *D* and *F*). Since SAS6 binding to STIL requires the STAN domain, data together support that STIL is the primary target of the ligase and SAS6 degradation by the ligase occurs in its STIL-associated form. In line with the effects on centrosomal levels of STIL and SAS6, Flag-FBXW7 expression also suppressed the supernumerary centriole defect induced by GFP-STIL overexpression under endogenous STIL depletion in G1/S synchronized HeLa cells. While ∼ 50% cells possessed three and more centrioles in the empty Flag-transfected cells, the same was reduced to ∼ 20% upon Flag-FBXW7 expression. As expected, elevated expression of GFP-STILΔSTAN did not cause centriole amplification, rather the majority of the cells contained two centrioles in either control Flag or Flag-FBXW7 expressed condition (Fig. 5*G*).

**Figure 6:**
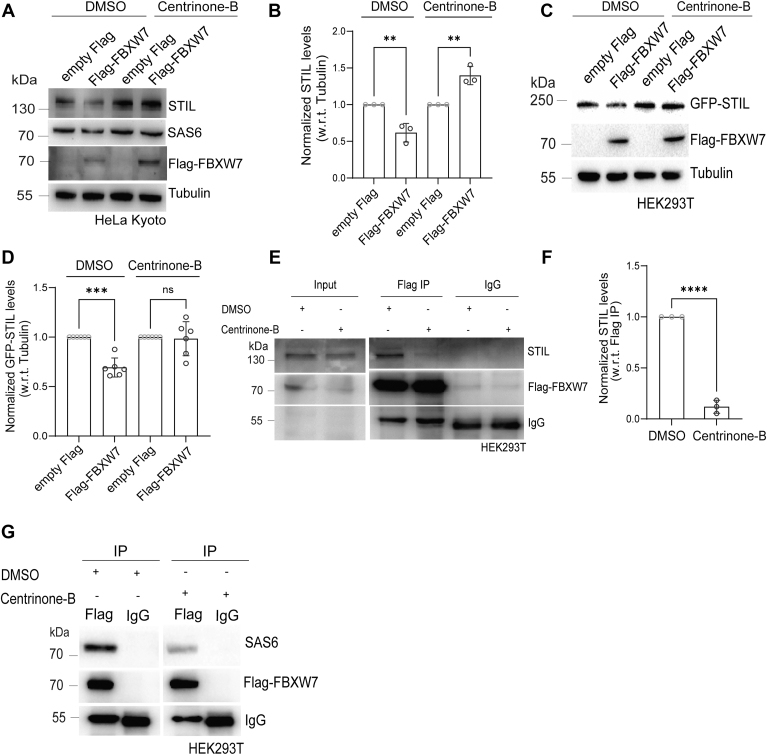
**FBXW7-mediated STIL and SAS6 degradation is dependent on Plk4 kinase activity.***A,* lysates of G1/S synchronized HeLa Kyoto cells transfected with either empty Flag or Flag-FBXW7 (48 h) and treated with Centrinone-B (100 nM) during the last 20 h were subjected to Western blot and probed for STIL and SAS6. *B*, quantification plot of STIL levels normalized with tubulin in empty Flag or Flag-FBXW7-expressed condition in absence and presence of Centrinone-B, N = 3, ∗∗*p* < 0.01 (One-way Anova). *C*, lysates of GFP-STIL together with empty Flag or Flag-FBXW7-expressed (48 h) G1/S synchronized HEK293T cells treated with Centrinone-B (100 nM) during the last 20 h were subjected to Western blot. *D*, quantification plot of GFP-STIL levels normalized with tubulin in empty Flag or Flag-FBXW7 expressed condition in absence and presence of Centrinone-B, N = 6, ∗∗∗*p* < 0.001 (one-way Anova). *E*, lysates of G1/S synchronized HEK293T cells expressed with empty Flag or Flag-FBXW7 in the absence or presence of Centrinone-B (100 nM) were subjected to Flag pulldown to assess the presence of associated endogenous STIL. *F*, quantification of STIL associated with Flag-FBXW7 in the lysates of cells expressed with Flag-FBXW7 in the absence or presence of Centrinone-B (100 nM). Data are mean ± S.D. (N = 3). ∗∗∗∗*p* < 0.0001 (Unpaired parametric *t* test). *G*, lysates of G1/S synchronized HEK293T cells expressed with empty Flag or Flag-FBXW7 in the absence or presence of Centrinone-B (100 nM) were subjected to Flag pulldown to probe for the presence of associated SAS6. STIL, SCL/TAL1-interrupting locus.

Plk4-mediated STIL phosphorylation in its STAN domain is required for centriolar recruitment of SAS6 and formation of the new centriole (45, 46). We asked if Plk4 also plays any role in FBXW7-mediated STIL regulation. HeLa Kyoto cells transfected with Flag-FBXW7 or empty Flag synchronized at G1/S were treated with Plk4 inhibitor, Centrinone-B (20 h) (47). Briefly, cells were exposed to Centrinone-B (100nM) for 20 h after 28 h of transfection of the exogenous empty Flag or Flag-FBXW7 plasmids. The level of Flag-FBXW7 expression over the endogenous FBXW7 was about ∼two fold (Fig. S3*C*). It was confirmed that Plk4 activity was inhibited upon Centrinone-B treatment with the expected overaccumulation of Plk4 at the centrosome (Fig. S3, *D* and *E*) and consequent reduction of STIL localization at the centrosome (Fig. S3, *D* and *F*) (47). While in the absence of Centrinone-B, STIL was markedly reduced under Flag-FBXW7 expression, the effect was much less pronounced in cells that were treated with Centrinone-B (100 nM) (Fig. 6, *A* and *B*). Similar nature of regulation was also observed for SAS6 (Fig. 6*A*). Corroborating with the effects on endogenous STIL, Centrinone-B treatment also suppressed Flag-FBXW7-mediated degradation of exogenously expressed GFP-STIL in HEK-293T cells (Fig. 6, *C* and *D*). Unlike the full-length GFP-STIL-overexpressed cells, the cells overexpressed with GFP-STILΔSTAN did not show significant change in its level in response to Flag-FBXW7 expression either under Centrinone-B treatment or in its absence (Fig. S4, *A* and *B*). GFP-STILΔSTAN also showed substantially less ubiquitination as compared to GFP-STIL WT as assessed from the immunoprecipitates of GFP-STIL *versus* GFP-STILΔSTAN from the lysates of HEK293T cells expressed with GFP-STIL or GFP-STILΔSTAN together with HA-Ub and Flag-FBXW7 (Fig. S4*C*). We next evaluated FBXW7-STIL interaction in the Plk4-inhibited condition. HEK293T cells transfected with Flag-FBXW7 for 48 h were synchronized at G1/S and treated with Centrionone-B (100 nM) prior to Flag pulldown. Association of STIL was markedly reduced in the Centrinone-B-treated cells as compared to cells in the absence of Centrinone-B (Fig. 6, *E* and *F*). Flag-FBXW7 association with SAS6 also showed marked reduction in the Flag pulldown samples from the lysates of G1/S synchronized HEK293T cells treated with Centrinone-B as compared to its absence (Fig. 6*G*).

### FBXW7 targets STIL via phosphodegron motif in STIL STAN domain

**Figure 7:**
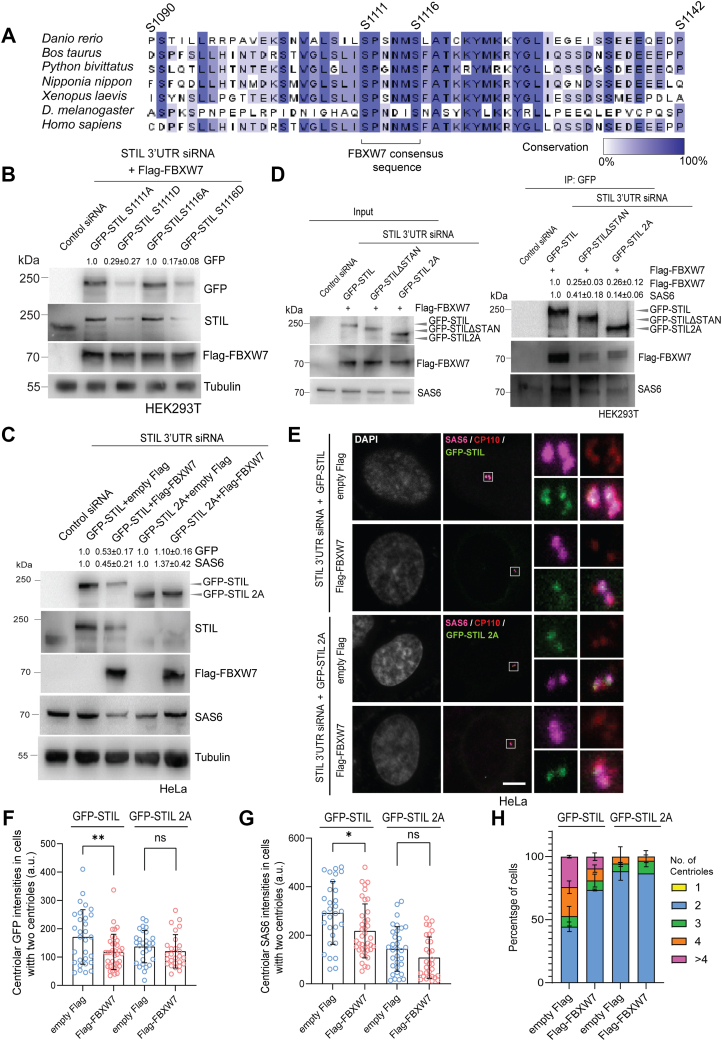
**FBXW7 targets STIL for degradation *via* putative phosphodegron motif in STIL STAN domain.***A*, amino acid sequence conservation of a major region of STIL STAN domain across different species. Ser residues S1090, S1111, S1116, and S1142 are known to be phosphorylated by Plk4. Ser 1111 (S1111) and Ser 1116 (S1116) in the FBXW7 consensus motif with sequence-(S/T)P-(X)3-(S/T/D/E/X)-(X = any amino acid) possess high conservation from *Danio rerio* to humans. *B*, lysates of HEK293T cells depleted of the endogenous STIL by 3′ UTR siRNA and expressed with GFP-STIL1111A, GFP-STIL1111D, GFP-STIL1116A, or GFP-STIL1116D together with Flag-FBXW7 (48 h) under G1/S synchronized condition were subjected to Western blot. N = 2. The intensity values of GFP-STIL proteins normalized to tubulin bands are shown. *C*, lysates of STIL 3′UTR siRNA-treated G1/S synchronized HEK293T cells expressed with GFP-STIL or GFP-STIL 2A and cotransfected with empty Flag or Flag-FBXW7 (48 h) were subjected to Western blot. Intensity values of SAS6 and GFP-STIL proteins normalized to tubulin bands as shown are based on three experiments. (Unpaired parametric *t* test). *D*, lysates of G1/S synchronized STIL 3′UTR siRNA-treated and GFP-STIL-, GFP-STILΔSTAN, and GFP-STIL 2A-overexpressed HEK293T cells cotransfected with Flag-FBXW7 (48 h) followed by treatment with MG-132 (6h) were subjected to immunoprecipitation (IP) by GFP-trap beads. The GFP-tagged STIL proteins and Flag-FBXW7 of input and the IP samples were probed by Western blot. Intensity values of Flag-FBXW7 and SAS6 normalized to the pulled-down GFP-STIL proteins are shown. N = 3, (Unpaired parametric *t* test). *E*, representative confocal images of GFP-STIL or GFP-STIL 2A expressed in STIL 3′UTR siRNA-treated HeLa cells cotransfected with empty Flag or Flag-FBXW7 (48 h) synchronized at G1/S and stained for SAS6 and CP110 (centriole marker). Scale bar = 5 μm. *F*, quantification plot of centrosome-localized GFP-STIL and GFP-STIL 2A levels in G1/S HeLa cells under expression of empty Flag or Flag-FBXW7. *G*, quantification plot of centrosome localized SAS6 levels in GFP-STIL *versus* GFP-STIL 2A-expressed G1/S HeLa cells under expression of empty Flag or Flag-FBXW7. Cells containing two centrioles were considered for the quantification in *F* and *G*. Data = mean ± S.D., n ∼70 cells for GFP-STIL and ∼35 cells for GFP-STIL 2A. ∗∗*p* < 0.01 and ∗*p* < 0.05 (Unpaired parametric *t* test). *H*, plot of centriole numbers in G1/S synchronized GFP-STIL or GFP-STIL 2A-expressed STIL 3′UTR siRNA-treated G1/S synchronized HeLa cells cotransfected with empty Flag or Flag-FBXW7. Data = mean ± S.D., n = ∼ 70 cells. STIL, SCL/TAL1-interrupting locus.

FBXW7 mediates ubiquitination of proteins by recognizing specific consensus phosphodegron motifs in its target substrate proteins. Amino acid sequence analysis revealed presence of a phosphodegron-like motif, with sequence -(S/T)P-(X)3-(S/T/D/E/X)- (S/T = Serine/Threonine; X = any amino acid) (48, 49, 50, 51, 52) in the STAN domain of STIL, with sequence S^1111^PSNMS^1116^ consisting of two serine residues, Ser 1111 and Ser 1116, both of which are conserved across several vertebrates (Fig. 7*A*). Both Ser 1111 and Ser 1116 are phosphorylated by Plk4, and moreover, phosphorylation of both the serine residues facilitates STIL–SAS6 interaction, centriolar recruitment of SAS6, and formation of new centriole (18, 26, 45, 53). We investigated the possible role of phosphorylation of these sites for FBXW7-mediated STIL degradation. To determine the relative roles of phosphorylation of these sites in STIL regulation, single phospho-resistant (S111A and S1116A) or phospho-mimicking (S111D and S1116D) mutants of STIL were generated and transfected together with Flag-FBXW7 in HEK293T cells depleted of endogenous STIL by STIL 3′UTR siRNA, and the levels of the mutant proteins were compared from the lysates of G1/S synchronized cells. Levels of both S1111D and S1116D STIL mutants were reduced upon Flag-FBXW7 expression as compared to their corresponding S to A mutant variants, supporting critical role of phosphorylation of both the sites in FBXW7-mediated STIL degradation (Fig. 7*B*). Effect of phospho-resistant mutation of both Ser 1111 and Ser 1116 (GFP-STIL 2A) was then compared with respect to WT GFP-STIL on their stability against FBXW7-induced degradation. HeLa cells depleted of endogenous STIL by 3′UTR siRNA with simultaneous expression of GFP-STIL (WT) or GFP-STIL 2A were transfected with Flag-FBXW7 or control Flag plasmid, and the levels of GFP-STIL proteins were assessed in G1/S synchronized condition. While the level of WT GFP-STIL was substantially reduced upon Flag-FBXW7 expression, GFP-STIL 2A level did not show detectable change upon Flag-FBXW7 expression as compared to its level in cells under control Flag expression (Fig. 7*C*). It is noticed that STIL antibody could not efficiently recognize the GFP-STIL 2A protein and were detectable clearly by GFP antibody. This could be due to an altered affinity of the STIL antibody, which is against the C-terminal region of STIL and near to the 2A mutation sites. Similar differences of GFP-STIL *versus* GFP-STIL 2A mutant stability were also observed in GFP-STIL- and GFP-STIL 2A-expressed cells without depletion of the endogenous STIL in HEK293T cells (Fig. S4, *D* and *E*). Interestingly, exogenously expressed Flag-FBXW7 failed to exert SAS6 degradation in cells with overexpressed GFP-STIL 2A mutant under endogenous STIL depletion; though it did so in the WT GFP-STIL-overexpressed cells under similar condition (Fig. 7*C*), suggesting that phosphorylated STIL is the main target of the ligase, and SAS6 degradation is induced, when it is associated with the phosphorylated STIL. Consistent with impaired regulation of STIL degradation, GFP-STIL 2A also showed markedly reduced interaction with Flag-FBXW7 as compared to WT GFP-STIL in cells under depletion of endogenous STIL with simultaneous expression of GFP-STIL or GFP-STIL 2A as revealed from the immunoprecipitates of the GFP-STIL proteins (Fig. 7*D*). A similarly diminished interaction was also evident between GFP-STILΔSTAN and Flag-FBXW7 in cells expressed with GFP-STILΔSTAN and Flag-FBXW7 under endogenous STIL depletion (Fig. 7*D*). Furthermore, SAS6 association with GFP-STIL 2A or GFP-STILΔSTAN was markedly reduced in either in the GFP-STIL 2A or GFP-STILΔSTAN-expressed cells as compared to that with WT GFP-STIL in the GFP-STIL-expressed cells (Fig. 7*D*). Previous study indicated that S1116 phosphorylation has less role in promoting SAS6 recruitment and centriole duplication (26). It is possible that S1116 site phosphorylation is more involved in FBXW7-mediated STIL degradation. We therefore looked into the relative levels of STIL S1116D as compared to the STIL S1116A mutant and the associated SAS6 in a size exclusion chromatography (see Experimental Procedures). The cell lysates of STIL S1116 D-versus S1116A mutant-expressed cells depleted of the endogenous STIL protein were run through a Superpose six column. The expression levels of the GFP-tagged STIL proteins which appear comparable to the endogenous STIL protein and the level of endogenous STIL depletion upon siRNA treatment are shown (Fig. S5*A*). A significantly lesser amounts of both GFP-STIL S1116D and its associated SAS6 were coeluted in the case of GFP-STIL S1116D-transfected cells as compared to GFP-STIL S1116A and SAS6 that were co-eluted through the column from the lysates of GFP-STIL S1116A-transfected cells (Fig. S5, *B*–*D*). This observation further supports that STIL S1116D form and its associated SAS6 are more susceptible to degradation as compared to the S1116A form of STIL and associated SAS6.

We next assessed the centriolar level of GFP-STIL 2A mutant at the centrosome upon Flag-FBXW7 expression in comparison with WT GFP-STIL. Endogenous STIL-depleted HeLa cells overexpressed with GFP-STIL or GFP-STIL 2A were transfected with Flag-FBXW7 or empty Flag, and the relative levels of centriole localization of the GFP-tagged STIL proteins were examined in the G1/S synchronized cells. As GFP-STIL overexpression induced supernumerary centrioles, the cells with two centrioles were considered for a better comparison of the centriolar levels of GFP-STIL *versus* GFP-STIL 2A. Under the control Flag expression, GFP-STIL 2A mutant was able to be recruited to the centrioles nearly to the similar level as of the WT GFP-STIL (Fig. 7, *E* and *F*). This is consistent with previous studies (26, 45). As anticipated, centriolar localization of GFP-STIL showed a substantial reduction upon Flag-FBXW7 expression; whereas, under similar condition, the centriolar level of GFP-STIL 2A did not exhibit significant change as compared to the empty Flag-expressed cells (Fig. 7, *E* and *F*, S5*D*). A similar regulation of centriolar SAS6 level was also apparent. Flag-FBXW7 expression considerably reduced its centriolar localization in GFP-STIL overexpressed cells but not in GFP-STIL 2A overexpressed cells (Fig. 7*G*). Phospho-deficient mutation also did not seem to affect FBXW7 overexpression-induced suppression of centriole duplication. Overexpression of WT GFP-STIL induced centriole amplification in cells with four or more centrioles, and the same was significantly suppressed upon Flag-FBXW7 overexpression (Fig. 7*H*). However, GFP-STIL 2A mutant overexpression failed to induce centriole amplification in the control empty Flag-expressed cells (Fig. 7*H*), likely because of its reduced ability to bind SAS6 (Fig. 7*D*) (26). Consistent with reduced ability of Flag-FBXW7 to bind to and degrade GFP-STIL 2A mutant, Flag-FBXW7 overexpression in the GFP-STIL 2A mutant-expressed cells did not further result in any further change in centriole number as compared those in the control Flag-transfected GFP-STIL 2A-expressed cells (Fig. 7*H*). The results together imply that phosphorylation promotes FBXW7-mediated STIL and its associated SAS6 degradation.

### Increased expression of FBXW7 is associated with a high degree of aneuploidy and with increased expression of STIL in cancer cells

**Figure 8:**
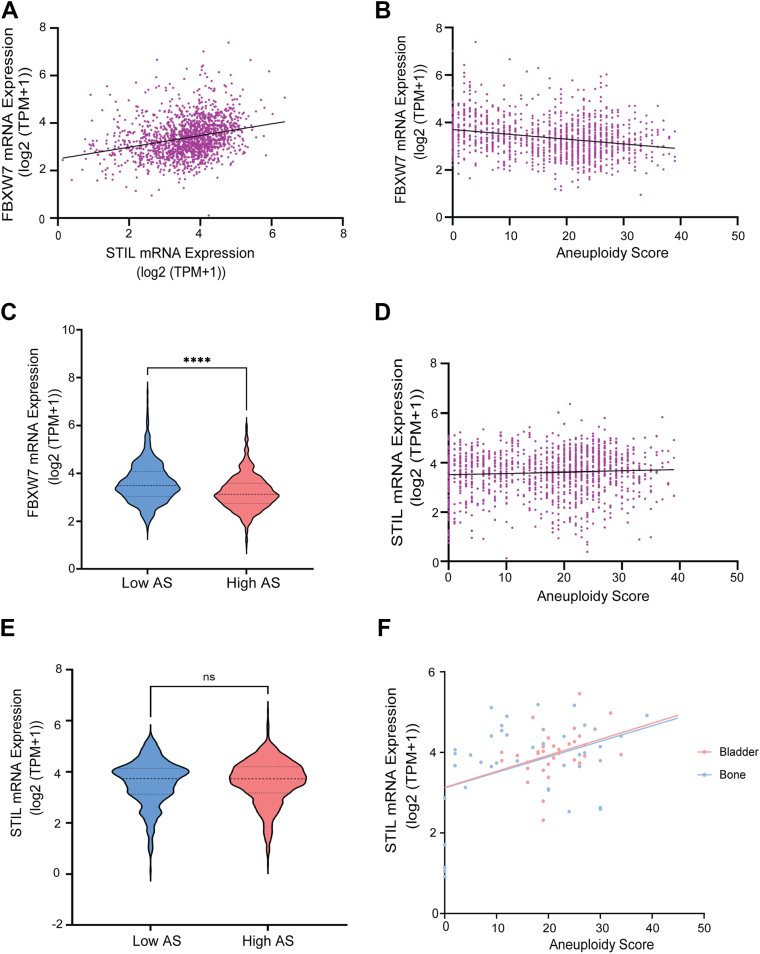
**Association of FBXW7 and STIL expression with aneuploidy across human cancer cell lines.** Data obtained from the Cancer Dependency Map (DepMap), version 24Q4 (Tsherniak *et al.*, 2017). *A*, FBXW7 and STIL expression are positively correlated at the mRNA level across cancer cell lines. Pearson’s correlation: r = 0.215, *p* < 0.0001. Linear regression coefficient: 0.201, *p* < 0.0001. *B*, FBXW7 mRNA expression is negatively correlated with aneuploidy scores across cancer cell lines. Spearman’s correlation: rho = −0.234, *p* < 0.0001. *C*, FBXW7 mRNA levels are significantly lower in cell lines with high aneuploidy scores compared to those with low aneuploidy scores. Sample sizes: n = 805 (high) and n = 498 (low). Two-sided *t* test: ∗∗∗∗*p* < 0.0001. Weighted *t* test: Cohen’s d = −0.254, *p* < 0.0001. Violin plots: central *bold dashed line* indicates the median; *lower* and *upper dashed lines* indicate the 25th and 75th percentiles, respectively. *D*, no significant correlation was observed between STIL mRNA expression and aneuploidy scores in a pan-cancer analysis. Spearman’s correlation: rho = 0.053, *p* = 0.0536. *E*, STIL mRNA levels do not differ significantly between high and low aneuploidy score groups. Two-sided *t* test: *p* = 0.5378. Violin plot conventions as in panel (*C*). *F,* positive correlation between STIL mRNA expression and aneuploidy scores observed within specific cancer types. Spearman’s correlations: bladder cancer: rho = 0.407, *p* = 0.02; bone cancer: rho = 0.316, *p* = 0.041. AS, aneuploidy scores; STIL, SCL/TAL1-interrupting locus.

Having established the mechanistic relationship between FBXW7 and STIL in regulating centriole duplication, we next examined whether FBXW7 expression and essentiality are associated with chromosomal instability and STIL expression in human cancers. To this end, we analyzed mRNA expression levels from 1517 human cancer cell lines using datasets from the DepMap portal (54, 55). While our mechanistic studies suggest that FBXW7 targets STIL for degradation, we found that FBXW7 mRNA expression was positively correlated with STIL mRNA expression across cancer cell lines (*p* < 0.0001) (Fig. 8*A*). This trend was also observed within individual cancer types and in a pan-cancer analysis when controlled for cancer type (Fig. S6*A*), suggesting the existence of feedback or compensatory mechanisms that maintain STIL expression in the presence of high FBXW7 levels. Consistent with the finding that FBXW7 loss promotes centriole amplification, we observed that FBXW7 mRNA levels were significantly lower in highly aneuploid, chromosomally unstable cell lines in comparison to near-diploid, chromosomally stable cell lines (*p* < 0.0001) (Fig. 8, *B* and *C*). This inverse association between FBXW7 expression and aneuploidy was also apparent within specific cancer types and in a pan-cancer analysis controlled for cancer type (Fig. S6*B*). Interestingly, although STIL mRNA levels did not show a significant correlation with aneuploidy scores in a pan-cancer analysis (*p* = 0.0536, *p* = 0.5378 respectively Figure 8, *D* and *E*), when stratified by cancer type, positive correlations between STIL expression and aneuploidy were evident in bladder/urinary tract, bone, and prostate cancers (Fig. 8*F*). Together, these results suggest that the functional interaction between FBXW7 and STIL is broadly relevant across cancer types. Moreover, the association of low FBXW7 expression with chromosomal instability and high aneuploidy supports our experimental findings, implicating FBXW7 as a crucial regulator of centriole homeostasis and genome stability in cancer.

## Discussion

Uncontrolled assembly of the template cartwheel structure made by STIL and SAS6 on the mother centriole is a key process through which overduplicated centrioles are generated. Level of STIL-SAS6 axis on the mother centriole requires to be maintained precisely to ensure that the cartwheel assembly and the subsequent new centriole assembly occur only once during each cell cycle. Here, we demonstrate that FBXW7 governs this process by targeted degradation of the STIL-SAS6 axis. We have shown that FBXW7-mediated STIL and SAS6 degradation is attenuated upon mutation of STIL phosphorylation sites, which abrogates STIL interaction with SAS6 (Fig. 7*D*) (26). Our data also have revealed that FBXW7 fails to induce SAS6 degradation in the STIL-depleted condition (Fig. 3*E*). Together, these findings substantiate the mechanism that STIL is the primary target of FBXW7, and degradation of SAS6 occurs in its STIL-associated form. FBXW7 overexpression markedly diminishes the centriolar levels of both endogenous STIL and its exogenously expressed variant under the endogenous STIL-depleted condition, suggesting that the causal connection between STIL and FBXW7 exists at the physiological STIL level. Further, such regulation is direct since absence of the substrate-binding domain of FBXW7 abolished FBXW7-mediated effect on STIL degradation and its centriolar level (Fig. 3). FBXW7 depletion leads to increased stabilization of STIL and SAS6 in the form of multiple foci at the centrosome by generating multiple mother-daughter centriole pairs. Further, our expansion microscopy imaging data in FBXW7 KO cells supported that such excess number of mother-daughter centriole pairs are generated owing to untimed activation of cartwheel assembly and the procentriole formation from the cartwheel prior to complete maturation of the parent centrioles (Fig. 2). The results, together, are in support of FBXW7’s involvement by maintaining the pool of STIL-SAS6 axis at a functionally optimal level on the mother centriole to maintain the correct centriole duplication frequency.

Upstream Plk4-mediated STIL phosphorylation in its STAN domain is crucial for STIL–SAS6 interaction and their corecruitment to the mother centriole leading to cartwheel assembly (18, 19, 26, 45). Interestingly, our study reveals that Plk4 activity is essential for FBXW7-mediated STIL-SAS6 degradation. Further, we have shown that STIL phospho-deficient mutation of key Plk4-targeting phosphorylation sites, Ser 1111 and Ser 1116 within a short-conserved motif in the STAN domain exerts resistance against FBXW7 overexpression-induced suppression of the centriolar levels of both STIL and SAS6 (Fig. 7). Such loss-of-function was evident in cells under depletion of endogenous STIL along with simultaneous expression of the phospho-deficient mutant substantiating a direct role of the phosphorylation. In further support, our molecular analyses data have shown that phosphorylation at both Ser 1111 and Ser 1116 promotes FBXW7-mediated STIL degradation, and the corresponding phospho-resistant mutation of either of the sites renders STIL less sensitive toward degradation (Fig. 7*B*). Our results therefore have uncovered a key molecular link of FBXW7 with Plk4-mediated STIL phosphorylation. Additionally, since the STIL - S^1111^PSNMS^1116^ motif bears close resemblance to the FBXW7 consensus phospho-degron motif and their phospho-resistant mutations of S1111 and S1116 impairs STIL–FBXW7 interaction (Fig. 7*D*), a plausible mechanism is that the phosphorylated STIL motif serves as a phosphodegron for the ligase. Altogether, it can be concluded that FBXW7 maintains centriole number homeostasis by targeting the centriole assembly-promoting phosphorylated form of STIL and its associated SAS6.

**Figure 9:**
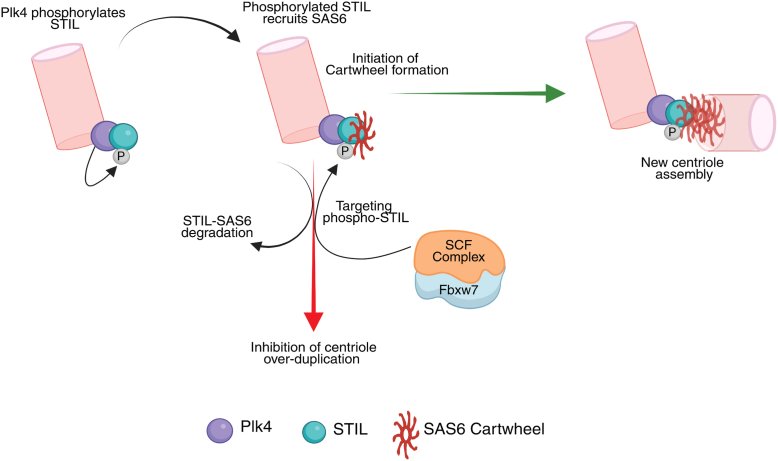
**A model depicting how FBXW7 regulates centriole duplication.** At the onset of G1/S transition, Plk4 phosphorylates STIL and facilitates centriolar STIL recruitment. Phosphorylated STIL and its binding protein SAS6 activates assembly of the cartwheel, from where the new centriole assembly is commenced. FBXW7 prevents the cartwheel assembly and new centriole generation by targeting phosphorylated STIL and its associated SAS6 and promoting ubiquitin-mediated degradation of STIL-SAS6 axis. STIL, SCL/TAL1-interrupting locus.

We have shown that siRNA-mediated depletion of FBXW7 led to increased stabilization of both STIL and SAS6 at the centrosome (Figs. 1, S1). Similarly, elevated expression of FBXW7 stimulates degradation of both the proteins and impairs their centriolar abundance (Figs. 3, S1). It is possible that degradation of both the proteins is coregulated by the ligase. In support of such mechanism, we have shown that deletion of the SAS6-interacting STAN domain renders both STIL and SAS6 resistant toward their degradation by exogenously expressed FBXW7 (Fig. 5*C*). In further support, exogenous FBXW7 expression affected centriolar GFP-STIL and SAS6 localization in cells with overexpressed GFP-STIL under the endogenous STIL-depleted background but failed to do so in cells overexpressed with the STAN domain-deleted STIL form (Fig. 5, *D*–*F*). Together, the findings are in support of the notion that FBXW7 targets STIL and its associated SAS6 for degradation. Our findings also reveal that Plk4 kinase activity plays an essential role in this process as Plk4 inhibitor substantially diminished FBXW7 overexpression-induced downregulation of both STIL and SAS6 levels (Fig. 6). STIL interacts with Plk4 through its central coiled-coil domain, and STIL binding to Plk4 further activates Plk4 kinase activity, which stimulates STIL phosphorylation in the STAN domain and subsequent association of the phosphorylated STIL with SAS6 (17, 18, 19, 26, 28, 45, 56). On the other side, we observed that exogenous FBXW7 overexpression suppresses centriolar level of the full-length STIL but minimally affects that of its STAN-deleted form (Fig. 5, *D* and *E*). The data together indicate that STAN is the key regulatory domain through which the centriolar STIL is targeted by the ligase. It is further supported by our data that FBXW7 fails to degrade the STAN domain-deleted form of STIL (Fig. 5*C*). It was also observed that FBXW7 fails to exert any effect on the centriolar localization of the phospho-resistant STIL 2A mutant form, in which the key Plk4 phosphorylation specific sites, Ser 1111 and Ser 1116, in the STAN domain are mutated to Ala (Fig. 7, *E* and *F*). Similar results were evident for centriolar localization of SAS6 as well (Fig. 7, *E* and *G*). The same mutation attenuates FBXW7-mediated STIL degradation (Fig. 7*C*), whereas the phospho-mimicking mutation of either of the two sites promotes the same (Fig. 7*B*). In a similar line, the phospho-deficient mutations of Ser 1111 and Ser 1116 greatly diminishes STIL binding to both FBXW7 and SAS6 (Fig. 7*D*). Therefore, it is logical to conclude that STIL degradation is induced by FBXW7 by targeting the Ser1111 and Ser1116-phosphorylated form of STIL. We have further shown that Ser1116 phosphorylation, though less critical for SAS6 targeting to centrosome and centriole duplication (26), promotes FBXW7-mediated STIL degradation (Fig. 7*B*). Taken together, the results support a model that FBXW7’s action on the STIL–SAS6 complex could interfere with the procentriole assembly process by facilitating degradation of the phosphorylated STIL and its associated SAS6 (Fig. 9).

What could be the physiological relevance of degradation of the STIL-SAS6 axis by FBXW7? It has been demonstrated previously in human cells that Plk4-mediated STIL phosphorylation activates formation of STIL–SAS6 complex, and further STIL and SAS6 are dependent on each other for their loading onto the mother centriole (25, 26). This relation appears to be conserved in other species as well (27, 57, 58). Very recent work in *C. elegans* also supported that STIL and SAS6 are corecruited to the mother centriole in the form of complex, and Plk4 kinase-mediated phosphorylation in STIL plays an essential role in stabilizing the complex on the mother centriole (24). Therefore, degradation of the STIL–SAS6 complex is likely a feasible mechanism for effective inhibition of unscheduled cartwheel assembly and centriole overduplication. Our results that Plk4 kinase activity is essential for STIL-SAS6 degradation by FBXW7 and Plk4-targeted phosphorylation in STIL STAN domain promotes FBXW7-mediated degradation of both STIL and SAS6 are in support of such a mechanism. What is its relevance with regards to regulation of other centriolar proteins, such as Plk4 by another SCF family ligase, βTrCP (FBXW1)? βTrCP targets Plk4 for degradation at G1 and G1/S (59, 60), whereas present data support that FBXW7 depletion does not affect Plk4 level at G1/S (Fig. S1). Previous studies in human cells also indicated that βTrCP colocalizes with the ‘ring’ structure of Plk4 but not with its ‘dot’ structure, onto which STIL gets recruited. Consequently, the ring-organized Plk4 is presumably targeted for destruction by βTrCP (60, 61) supporting the mechanism that βTrCP does not target the fraction of Plk4 localized at the cartwheel assembly site. Though the ultrastructural details of centriolar FBXW7 localized with respect to STIL–SAS6 complex are yet to be elucidated, our data on FBXW7’s ability to target the two proteins in the complex form are in favor of its action at and near the cartwheel-emerging site.

Our analysis of a large-scale cancer cell line dataset further supports the functional link between FBXW7 and STIL uncovered in our experimental work. FBXW7 mRNA levels are lower in highly aneuploid cell lines and negatively correlated with aneuploidy scores. While FBXW7 acts as a negative regulator of STIL protein stability, their mRNA expression levels were found to be positively correlated across the cancer cell lines. This apparent paradox may simply reflect the expression of both genes in dividing cells, or it might suggest a compensatory feedback mechanism, whereby increased degradation of STIL due to high expression of FBXW7 is counterbalanced by transcriptional upregulation of STIL (Fig. 8). Therefore, the mRNA coexpression is not at odds with the protein-level antagonism.

Importantly, our findings also reveal that FBXW7 expression is inversely associated with aneuploidy and chromosomal instability across cancer cell lines. Given that FBXW7 limits centriole overduplication, its reduced expression likely facilitates the generation of supernumerary centrioles, contributing to mitotic errors and ongoing chromosomal mis-segregation, potentially explaining the observed association. In contrast, STIL expression showed only cancer type–specific associations with aneuploidy, suggesting that its contribution to chromosomal instability may depend on lineage-specific regulatory networks or selective pressures.

In summary, we have demonstrated that FBXW7 controls centriole amplification by restraining the centriolar level of STIL and SAS6 and inducing unscheduled assembly of cartwheel at prematured stage of the parent centrioles. The findings that Plk4 kinase-targeted phosphorylation in the SAS6-interacting STAN domain in STIL is a key determining factor for such control imply that FBXW7 governs the process by depleting the STIL–SAS6 complex from the procentriole assembly site. The ultrastructural details of the localization and dynamics of FBXW7 during the STIL–SAS6 complex-driven cartwheel assembly would enable in the future to further understand the temporal regulation, if any, in the process. As FBXW7 is mutated in numerous cancers (36, 37, 38, 39, 40) impacts of the mutations on STIL–SAS6 complex stability and cartwheel assembly will also be important to uncover.

## Experimental procedures

### Antibodies and reagents

Dulbecco’s modified Eagle’s medium, Minimal Essential Medium (Opti-MEM), and fetal bovine serum were purchased from Thermo Fisher Scientific. Thymidine, DAPI, MG132, and EGTA were procured from Sigma Aldrich. Centrinone-B was obtained from Med ChemExpress. GFP trap beads (gt-20) were obtained from Chromotech (Germany). Rabbit polyclonal antibody against FBXW7 (#ab12292, IF-1:500, WB-1:500) and STIL (#ab89314, WB-1:1000) were obtained from Abcam. Mouse monoclonal antibody against tubulin (#T5168, WB-1:2000), FLAG M2 (#F1804, IF-1:500, WB-1:2000), and HA (#ab18181, WB-1:1000) were procured from Abcam. Mouse polyclonal antibody to Centrobin (#ab70448, IF-1:500) was obtained from Abcam. Mouse monoclonal GFP antibody (#632381, WB-1:1000) was obtained from Clontech, Takara. Mouse monoclonal antibodies for detection of Centrin-2 (20H5 04–1624, IF-1:500) and Plk4 (#MABC544, IF-1:50) were obtained from Merck Millipore. Rabbit DYKDDDDK tag Polyclonal antibody, which binds to Flag tag epitope (#20543-1-AP, IF-1:350, WB-1:2000), SAS6 (#21377-1-AP, IF-1:50), and CP110 (12780-1-AP, IF-1;500) were procured from Proteinech. Rabbit polyclonal antibody against STIL (#A302–442A, IF-1:500) was obtained from Bethyl laboratories. Mouse monoclonal antibody to SAS6 (#sc-376836, WB-1:1000) was obtained from Santa Cruz Biotechnology, Inc. Alexa fluor–conjugated donkey anti-mouse 488 and anti-rabbit 568 secondary antibodies were obtained from Invitrogen. Anti-mouse Cy5 and HRP-conjugated secondary antibodies were obtained from Jackson Immuno Research.

### Cell culture, siRNA, and plasmid transfection

HeLa Kyoto cells were obtained from Sachin Kotak, Indian Institute of Science (IISc), Bangalore (originally provided by Daniel Gerlich, IMBA). HEK293T, HeLa, and U2OS cells were originally obtained from ATCC. HEK293T, HeLa, U2OS, or HeLa Kyoto cells were grown in a humidified environment with 5% CO2 using Dulbecco’s modified Eagle’s medium supplemented with fetal bovine serum (10%), L-glutamine (2mM), sodium bicarbonate (1.5mg/ml), penicillin (100 ng/ml), and streptomycin (100 ng/ml). For depletion of desired protein, single siRNA was used. FBXW7 WT (FBXW7 ^+/+^) and homozygous FBXW7 ^−/−^ KO DLD-1 cell lines were obtained from B. Vogelstein lab, HHMI, Johns Hopkins and were cultured in McCoys5A medium supplemented with 10% fetal bovine serum, 2 mM L-glutamine and 100 mg/ml penicillin, streptomycin. For depletion of endogenous FBXW7, 60% confluent HeLa Kyoto cells were transfected with single FBXW7 siRNA (5′ ACCUUCUCUGGAGAGAGAAAUGC-3′) from Dharmacon. For the control experiment, control siRNA (5′-UCUAUAUCAUGGCCGACAA-3′) was used from Dharmacon. Methods used for transfection by SMARTpool siRNA (cat #L-004264–00, On-target plus SMART pool Human FBXW7 and Control (cat#D-001810–10–05, Non -Targetting pool from Dharmacon) were similar to that of single siRNA treatment. For depletion of endogenous β-TrCP, 60% confluent HeLa Kyoto cells were transfected with single β-TrCP siRNA (5′-GUGGAAUUUGUGGAACAU-3′) obtained from Eurogentec. Cells were synchronized in G1/S by single or double thymidine (2 mM) prior to collection for lysis or imaging (62). Cell lysis was performed using lysis buffer (20 mM Tris–HCl, pH 7.4, 0.1% Triton X-100, 50 mM NaCl, and 10 mM EGTA) supplemented with phosphatase inhibitor 2 and 3 and protease inhibitor (Sigma). For depletion of endogenous STIL with simultaneous expression of exogenous STIL plasmids, cells were transfected with STIL plasmids after 8h of treatment with STIL 3′-UTR siRNA (5′-GUUUAAGGGAAAAGUUAUU-3′) (Dharmacon). Lipofectamine 3000 (Invitrogen) or Polyethylenimine (Polysciences) was used for transfection of plasmid DNA while Lipofectamine RNAiMAX (Invitrogen) was used for transfection of siRNA.

### DNA constructs

Flag-tagged human FBXW7 (β-form) was originally obtained from Bruce E Clurman, Fred Hutchinson Cancer Research Center. It was further used as a template to generate Flag FBXW7-ΔWD (1–249) construct. FBXW7 siRNA-resistant construct was generated by site-directed mutagenesis using Flag-tagged human FBXW7 plasmid as template. EGFP STIL full-length (pENTR/D-TOPO_STIL_1–1287) plasmid was obtained from Eric A. Nigg lab, University of Basel, Switzerland. EGFP STIL C1 construct was made by PCR amplification of EGFP STIL FL from pENTR/D-TOPO_STIL_1-1287 as template and cloned into pEGFP C1 vector (provided by Dr Sandhya Ganesan, IISER Thiruvananthapuram) having a CMV promoter using infusion cloning. FBXW7 siRNA resistant construct was generated by site-directed mutagenesis using Flag-tagged human FBXW7 plasmid as template. EGFP STILΔSTAN C1 (Δ1052–1148) was generated by overlap PCR amplification of EGFP STIL FL and cloned into pEGFP C1 vector using infusion cloning. GFP-STIL 2A, STILS1111D, S1111A, S1116D, and S1116A mutants were generated by site-directed mutagenesis using EGFP STIL full-length as template.

### Co-immunoprecipitation and immunoblotting

Cells were lysed using lysis buffer (20mM Tris-HCl, pH 7.4, 0.1% Triton X-100, 50 mM NaCl, 10mM EGTA) supplemented with phosphatase inhibitors 2 and 3 and protease inhibitor (Sigma). Cell lysates were incubated with antibodies as specified for immunoprecipitation and then incubated with protein-A/G-agarose beads. Beads were washed with lysis buffer and then boiled in SDS-PAGE sample buffer prior to immunoblot analysis. All the immunoblots were developed in the ChemiDoc XRS + imaging system from Bio-Rad, and the images were processed by Image Lab 5.0 BioRad or Quantity one software. Quantity one software was used to perform raw data quantification and to obtain corresponding mean intensity values of the protein bands. Background intensities were obtained from regions adjacent to the protein bands and were subtracted from the total intensities of protein bands to obtain the net intensities of the bands. The net intensity values were normalized with respect to net intensities of the corresponding loading control. The data were plotted and analyzed using GraphPad Prism 9 software.

### Ubiquitination assay

HEK293T cells were transfected with HA-Ub and respective GFP-STIL constructs followed by empty Flag or Flag-FBXW7 transfection 6 h. After 36 h, cells were treated with thymidine (2 mM) alone for 12 h followed by MG132 (25 μM) for another 6 h before harvesting. GFP-STIL protein was then immunoprecipitated from the lysate of cells by using GFP trap beads (63). Samples were probed for GFP-STIL using GFP antibody. To visualize the ubiquitination bands properly, the immunoprecipitation samples of same volume were separately run in a 6% SDS gel and immunoblotted with the HA-antibody. STIL ubiquitination was quantified from the HA-Ub region normalized with respect to the intensity of GFP-STIL band.

### PLK4 inhibition by Centrinone-B

HeLa Kyoto cells were treated with 100nM of Centrinone-B after 20 h of transfection of the plasmids and then synchronized by single thymidine (2 mM) treatment for 20 h prior to collection for cell lysis (47).

### Immunofluorescence microscopy and image analyses

HeLa or HeLa Kyoto cells fixed in ice-chilled methanol were washed with PBS containing 2% bovine serum albumin and 0.5% Triton X-100. Cells were then incubated with respective primary antibodies for 3 h at room temperature or overnight at room temperature followed by incubation with secondary antibodies (Alexa 488 anti-mouse, Alexa 568 anti-rabbit and Cy5-conjugated anti-mouse, 1:1000) for 1 h at room temperature. Cells were incubated with DAPI (10μM, from Sigma) for 1 min and mounted on the coverslips by Prolong Gold. The images were captured by 63 × (1.4 N A.) oil-immersion objective in Leica SP 5 or Zeiss LSM 880 or Olympus FV3000 confocal microscope.

The image analysis and intensity adjustment were performed using Leica Application Suite Advanced Fluorescence Lite 2.8.0 software or ZEN BLUE software or ImageJ Fiji. Image contrast was adjusted by changing the LUT values for better visibility. Raw confocal images were quantified for individual centriolar intensities across Z or from projection images. Imaging parameters were kept the same in control *versus* treated conditions. For intensity measurements, the total pixel intensity of the regions of interest covering the whole centrosome area was measured. Background intensity was measured from a region adjacent to centrosome and subtracted from the centrosome intensities. For counting centriole number, the centriolar signals corresponding to the marker, and wherever relevant, the desired protein were counted. All graphs were plotted and analyzed for statistical significance by unpaired parametric *t* test using GraphPad Prism9.

### Expansion microscopy

Cells grown on coverslips were fixed in a solution of formaldehyde/acrylamide in PBS for 5h at 37 °C incubator, after rinsing with the cell extraction buffer (10mM K-PIPES, 0.3 M Sucrose, 0.1M NaCl, 3mM MgCl_2_, 10mM EGTA, 0.5% Triton X-100, and ddH_2_O). A monomer solution composed of 19% sodium acrylate, 10% acrylamide, 0.1% bis-acrylamide, 10× PBS, 10% APS, and 10% TEMED was added to a parafilm kept in a precooled chamber on ice. The coverslip with the fixed cells was quickly placed onto the parafilm with cells facing toward the monomer solution. Gelation proceeded for 5 min on ice, and then the samples were incubated at 37 °C for 1 h. Coverslips with the gels were then transferred to 2 ml of denaturation buffer (200 mM SDS, 200 mM NaCl, and 50 mM Tris in ddH_2_O) in a 6-well plate for 15 min at room temperature with 400 rpm gentle rotation. Gels were then removed from the coverslips and placed in a tube filled with 1.2 ml denaturation buffer and incubated at 95 °C for 1 h 30 min. After denaturation, the gels were placed in a tray filled with ddH2O for the first expansion. Water was exchanged after 1 h at room temperature, and then, the gels were incubated overnight in ddH2O. Next day, measurements were taken of the expanded gels using the Vernier scale (for expansion factor calculation). After that, gels were allowed to shrink by placing it in 1× PBS for 15 min. Gels shrank back to half their expanded size. A quarter of the gel was cut precisely and that chunk was used for antibody incubation. Incubation with primary antibodies (acetylated tubulin, 1:1000 and STIL, 1:500 diluted in 1% BSA in 1× PBS) was carried out at 37 °C for 3 h, with gentle shaking. Gels were then washed with PBS with 0.1% Tween 20, three times for 10 min on rocker shaker at room temperature and subsequently incubated with secondary antibody (Donkey anti-Rabbit 568 and Donkey anti-Mouse Cy5) solutions for 2h 30 min at 37 °C with gentle shaking. Sixty microliter of 10 μM DAPI was also added to the same solution separately for each well. Finally, the gel pieces were again placed in ddH2O-filled trays for expansion, and a small chunk was cut with a scalpel before proceeding for imaging in a μ-35 mm Ibidi dish (Ibidi).

### Size-exclusion chromatography

Lysates of the GFP-STIL-S1116A or GFP-STIL-S1116D expressed HEK-293T cells depleted of endogenous STIL were loaded onto a Superose 6 10/300Gl column (ÄKTA 10, GE Healthcare), pre-equilibrated with buffer (30mM Tris, 150mM NaCl, pH 7.4) at 4 °C. Following the injection of the lysate samples, 100μl of eluted protein fractions were collected and subjected to 90% (−80 °C) ethanol precipitation followed by denaturation with sample buffer. The samples were run in SDS-PAGE followed by Western blot. Protein markers, namely thyroglobulin (669 kDa), ferritin (440 kDa), alcohol dehydrogenase (82 kDa), conalbumin (75 kDa), albumin (66 kDa), ovalbumin (43 kDa), and carbonic anhydrous (29 kDa) were also run to obtain the standard molecular weight *versus* elution volume plot and subsequent determination of the size of the eluted GFP-STIL and SAS6 proteins by extrapolation. The GFP-STIL mutant and SAS6 were co-eluted at ∼ 16 ml (size 240 KDa), referring to the complex formation.

### Statistical analysis

Data presented are mean ± SD (standard deviation). Unpaired parametric *t* test or one-way ANOVA followed by Tukey’s multiple comparison test was used for statistical analysis. All graphs were plotted and analyzed for statistical significance in GraphPad Prism9. The figures were organized by Adobe Illustrator.

### Cancer cell line data analysis

Aneuploidy scores (ASs) and mRNA expression data for human cancer cell lines were obtained from the DepMap 24Q4 release (https://depmap.org/portal/) (54, 55). Cell lines were stratified into low and high aneuploidy score groups based on the median AS value. Differential gene expression between the two groups was assessed using a two-sided Student’s *t* test. Statistical analyses were conducted in RStudio using standard functions, in the DepMap data explorer portal and in GraphPad Prism 10.

To account for lineage as a potential confounder, inverse probability of treatment weighting was applied (64, 65), and both weighted t-tests and weighted Pearson correlations were performed in R. The t-tests and weighted analyses were conducted based on a binary stratification of samples into “high aneuploidy score” and “low aneuploidy score” groups, defined as those with aneuploidy scores above or below the cohort median, respectively. Additionally, linear regression models were fitted with AS and lineage as covariates to further control for lineage effects.

## Data availability

All relevant data are available.

## Supporting information

This article contains supporting information.

## Conflict of interest

U. B.-D. received consulting fees from Accent Therapeutics. The other authors declare that they have no conflict of interest.
