# Supplementary material for: FBXW7 E3 ligase prevents centriole overduplication by degrading the Plk4 phosphorylated STIL-SAS6 cartwheel assembly
Source: J Biol Chem. 2025 Dec 24;302(2):111104. doi: 10.1016/j.jbc.2025.111104 (PMC12858352; doi:10.1016/j.jbc.2025.111104)
Supplement: Supporting Information [file mmc1.docx]

**FBXW7 E3 ligase prevents centriole overduplication by degrading the Plk4 phosphorylated STIL-SAS6 cartwheel assembly**

Ushma Anand, Amit Bloomberg, Pradip Bhattacharjee, Swarnendu Mukhopadhyay, Binshad Badarudeen, Shivani Ramakrishnan, Uri Ben-David, Tapas K. Manna*

**Supplementary Data**

**
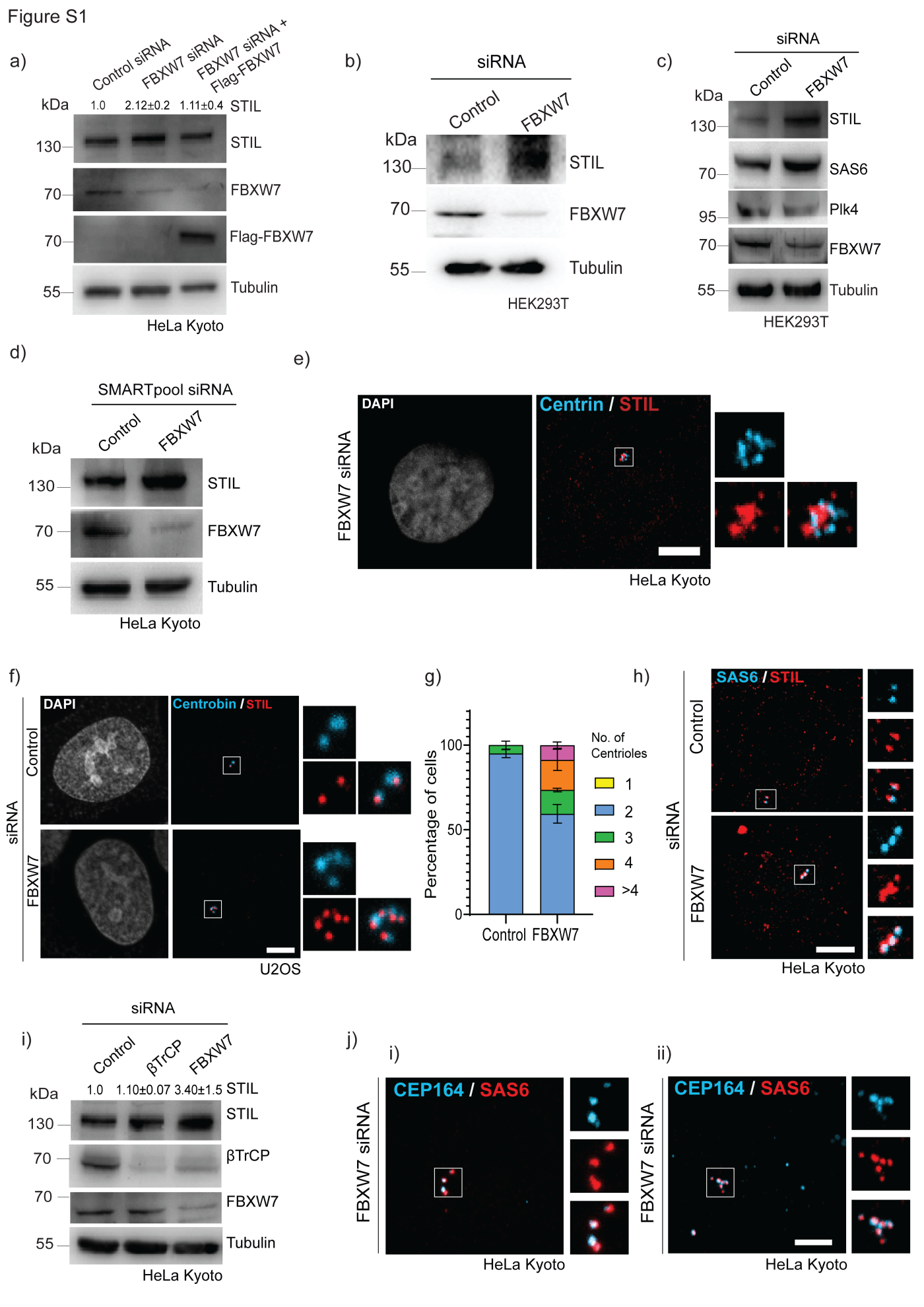
**

**Figure S1.** FBXW7 regulates stabilities of STIL and SAS6.

a. FBXW7 siRNA-treated HeLa Kyoto cells expressed with siRNA-resistant Flag-FBXW7 shows rescue of endogenous STIL level comparable to the level of control cells. Only FBXW7 siRNA-treated cells show increased level of endogenous STIL. Band intensities are mean ± S. D. (Unpaired parametric t-test). b. FBXW7 depletion induces stabilization of STIL in HEK293T cells. Lysates of HEK293T cells after transfection with control or FBXW7 siRNA followed by G1/S synchronization were probed for STIL and FBXW7 by Western blot. Tubulin was probed as control. c. Lysates of HeLa Kyoto cells after transfection with control or FBXW7 siRNA followed by G1/S synchronization were probed for SAS6, STIL, Plk4 and FBXW7. d. FBXW7 depletion by SMARTpool siRNA induces stabilization of STIL in HeLa Kyoto cells. Lysates of HeLa Kyoto cells after transfection with control or FBXW7 SMARTpool siRNA followed by G1/S synchronization were probed for STIL and FBXW7 by Western blot. Tubulin was probed as control. e. Additional set of representative confocal images of G1/S synchronized HeLa Kyoto cells depleted of FBXW7 by siRNA showing STIL localization as multiple foci as supernumerary centrioles (>4). Centrin-2 was stained as a centriole marker. Scale bar = 5 μm. f. Representative confocal images of U2OS cells after transfection with control or FBXW7 siRNA followed by G1/S synchronization and stained for Centrobin and STIL. Scale bar = 5 µm. g. Plot of percentage of cells with different number of centrioles in control vs. FBXW7 siRNA condition in U2OS cells is shown. Data plotted are from two sets of experiments. h. Representative confocal images of HeLa Kyoto cells after transfection with control or FBXW7 siRNA followed by G1/S synchronization and stained for SAS6 and STIL. Scale bar = 5 µm. i) Lysates of HeLa Kyoto cells after transfection with FBXW7 or β-TrCP siRNA followed by G1/S synchronization were probed for STIL and FBXW7 by Western blot. Tubulin was probed as control. Intensity values ± S. D. of STIL and FBXW7 normalized with respect to tubulin are shown based on three experiments(Unpaired parametric t-test). j) Additional set of representative confocal images (i, ii) of G1/S synchronized HeLa Kyoto cells treated with FBXW7 siRNA (48 h) stained for CEP164 and SAS6. Scale bar = 5 μm.

**
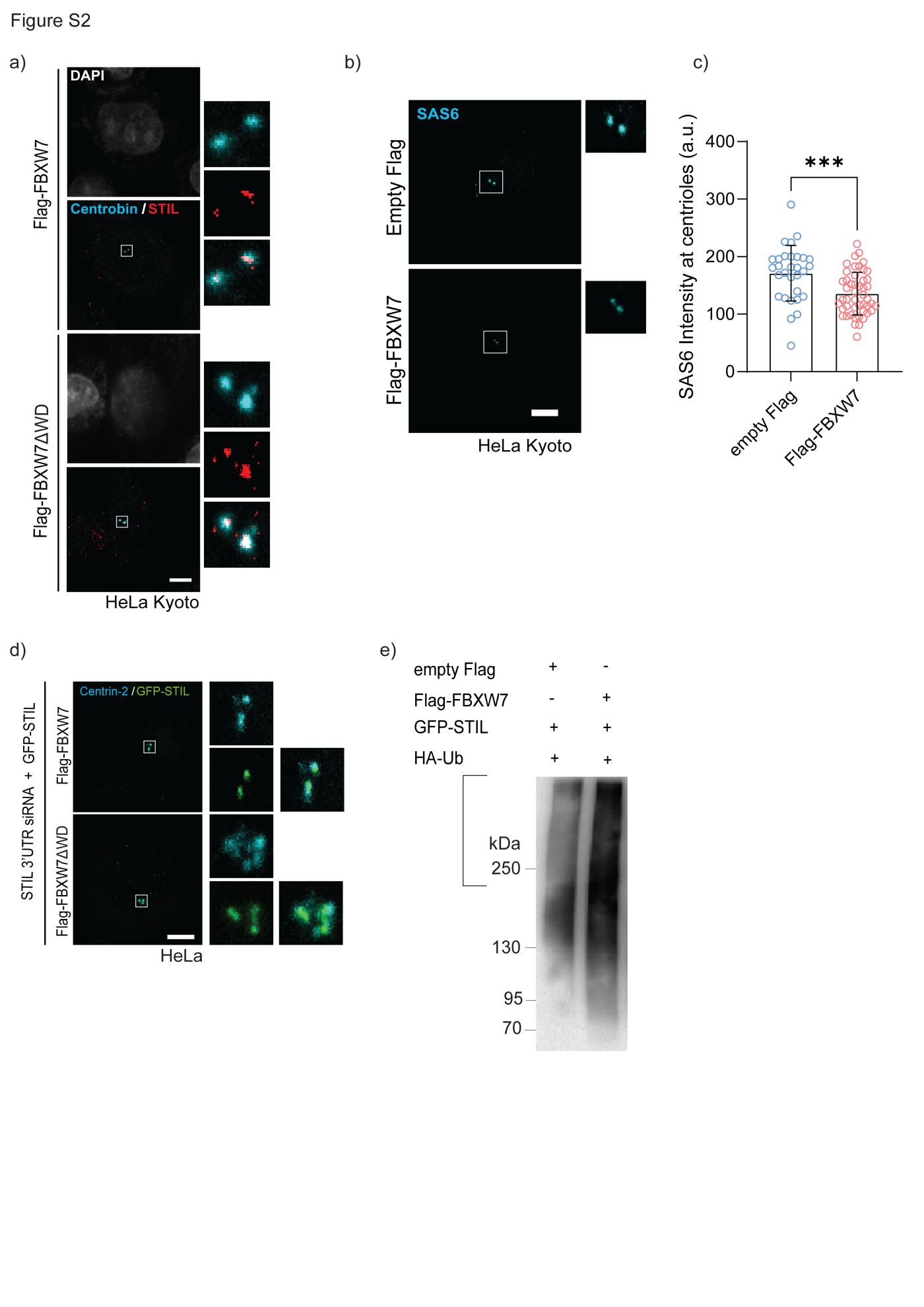
**

**Figure S2.** Overexpression of FBXW7 leads to reduction of STIL level and suppresses centriole amplification.

a. Additional set of representative confocal microscopy images of HeLa Kyoto cells expressed with Flag-FBXW7 or Flag-FBXW7ΔWD were stained for STIL and Centrobin under G1/S synchronized condition. Images of cells with centrioles (two Centrobin foci) are shown to visualize the differences of centriole-localized STIL intensity. b. Flag-FBXW7 overexpression results in significant loss of SAS6 from the centrioles in G1/S synchronized HeLa Kyoto cells. c. Plot of SAS6 intensity at centrosome in cells over-expressed with control or Flag-FBXW7. Cells containing two centrioles were considered for quantification. n= 30 cells each, ***p <0.001 (Unpaired parametric t-test). d. Additional set of representative confocal images of HeLa cells over-expressed with GFP-STIL under depletion of endogenous STIL by STIL 3`UTR siRNA and expressed with Flag-FBXW7or Flag-FBXW7ΔWD and stained with GFP and Centrin-2 antibody. GFP-STIL over-expression-induced supernumerary centriole (>2) defect was suppressed upon over-expression of Flag-FBXW7, but not of Flag-FBXW7ΔWD. e. The whole section of the HA-Ub stained immunoblot with reference to Figure 4e is shown. Samples were run in a 6% SDS gel and immunoblotted with the HA-antibody. Since GFP-STIL migrates at ~170 kDa size, the regions from and above this size of the HA-Ub -stained band were considered for quantification in Figure 4f.


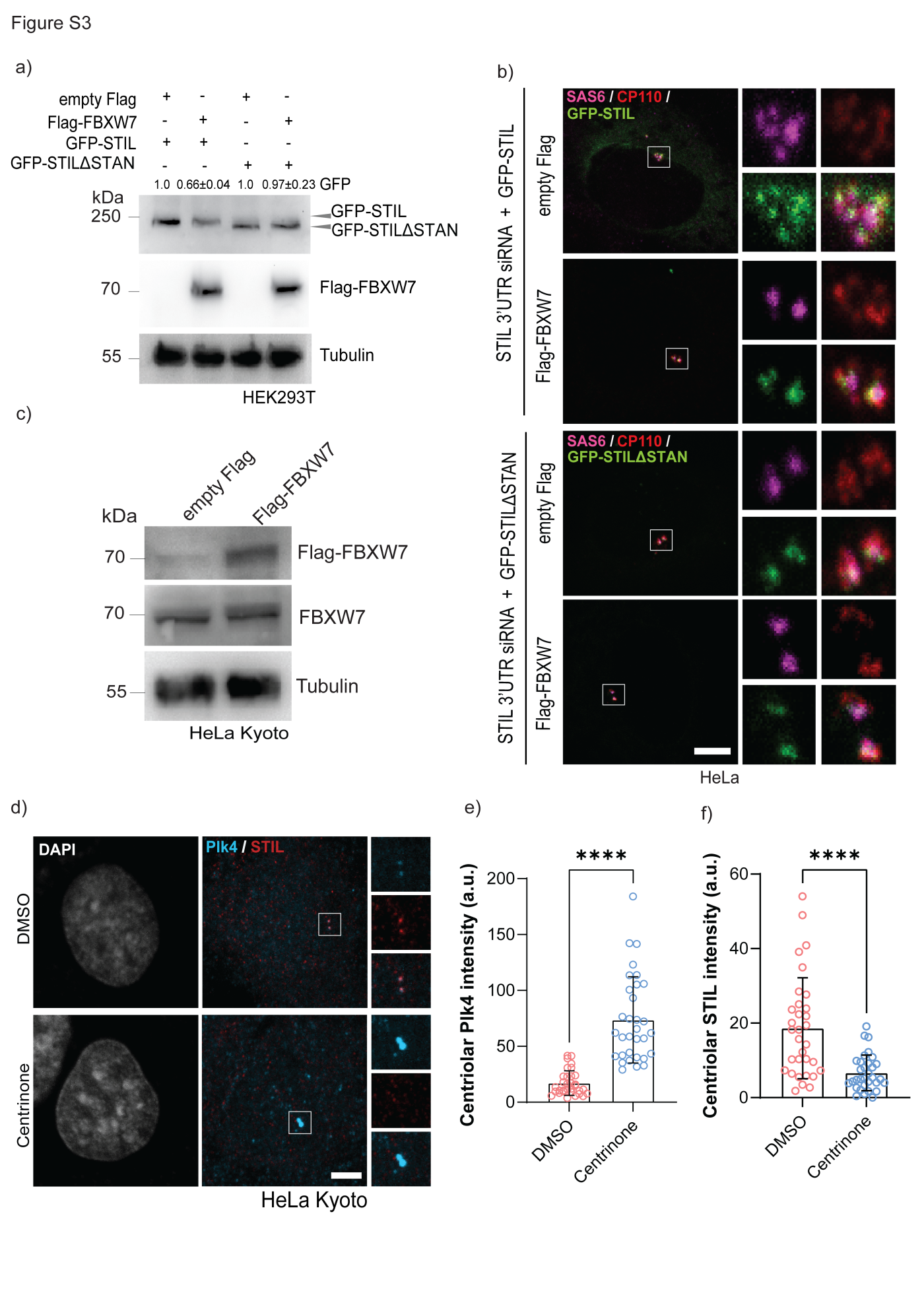


**Figure S3.** STAN domain of STIL is critical for FBXW7-mediated STIL degradation.

a. Lysates of HEK293T cells expressed with GFP-STIL and GFP-STILΔSTAN together with empty Flag or Flag-FBXW7 (48 h) under G1/S synchronized condition were subjected to Western blot. Intensity values of the GFP-STIL proteins normalized with respect to tubulin are shown based on three experiments (One-way Anova) b. Additional representative confocal images of GFP-STIL or GFP-STILΔSTAN-expressed STIL 3’ UTR siRNA-treated HeLa cells transfected with empty Flag or Flag-FBXW7 were synchronized at G1/S and stained for SAS6, CP110 (as marker) and GFP-STIL proteins. Scale bar = 5 μm. c. Lysates of G1/S synchronized HeLa Kyoto cells were transfected with empty Flag or Flag-FBXW7 and probed with FBXW7 and Flag antibody to assess the level of expression of Flag-FBXW7 over the endogenous protein. d. Representative confocal images of G1/S synchronized HeLa Kyoto cells treated with 100nM Centrinone-B or DMSO and stained for Plk4 and STIL. DNA was stained with DAPI. Scale bar = 5 µm. Inhibition of Plk4 kinase activity inhibition by Centrinone-B led to over-accumulation of Plk4 and reduced STIL levels at centrioles. e. Plot of Plk4 intensity at centrosomes in cells upon treatment with DMSO or Centrinone-B (100 nM). n= ~30 cells each, ****p <0.0001 (Unpaired parametric t-test). Data = mean ± S.D. f. Plot of STIL intensity at centrosomes in cells upon treatment with DMSO or Centrinone-B (100 mM). n= ~30 cells each, ****p <0.0001(Unpaired parametric t-test). Data = mean ± S.D.

**
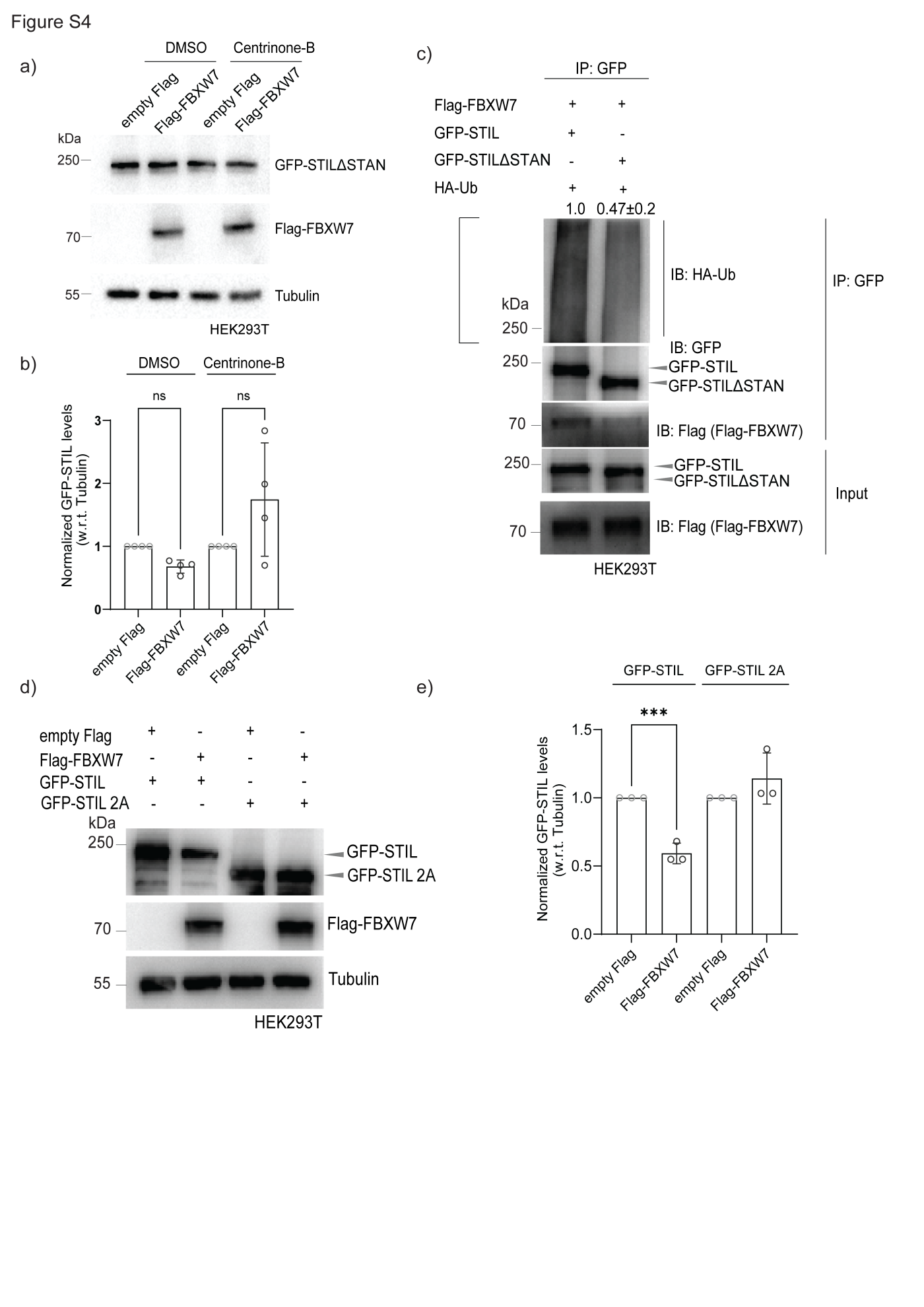
**

**Figure S4.**  Phosphorylation in STIL STAN domain at Plk4-targeted sites is critical for FBXW7-mediated STIL degradation.

a. Lysates of HEK293T cells over-expressed with GFP-STILΔSTAN together with empty Flag or Flag-FBXW7 (48 h) and treated with Centrinone-B (100 nM) during the last 20 h under G1/S synchronized condition were subjected to Western blot. b. Quantification plot of GFP-STILΔSTAN levels normalized with tubulin in empty Flag or Flag-FBXW7-expressed condition in the absence and presence of Centrinone-B as of a. N = 4 (One-way Anova). c. Lysates of GFP-STIL- or GFP-STILΔSTAN-over-expressed HEK293T cells co-transfected with HA-Ub and Flag-FBXW7 for 12 h followed by G1/S synchronization by thymidine (18 h) and MG-132 treatment (last 2 h), were subjected to immunoprecipitation by GFP trap beads. The samples were probed for the ubiquitinated proteins by HA antibody. Intensity values of HA-Ub proteins in the selected region as shown are based on normalization to GFP-STIL pulldown proteins (Unpaired parametric t-test). d. Lysates of HEK293T cells expressed with GFP-STIL or GFP-STIL 2A and co-transfected with empty Flag or Flag-FBXW7 (48 h) synchronized at G1/S were subjected to Western blot (representative of 3 experiments) e. Quantification plot of GFP-STIL and GFP-STIL 2A levels normalized with tubulin in empty Flag or Flag-FBXW7 expressed condition, N=3, ***p < 0.001 (One-way Anova).


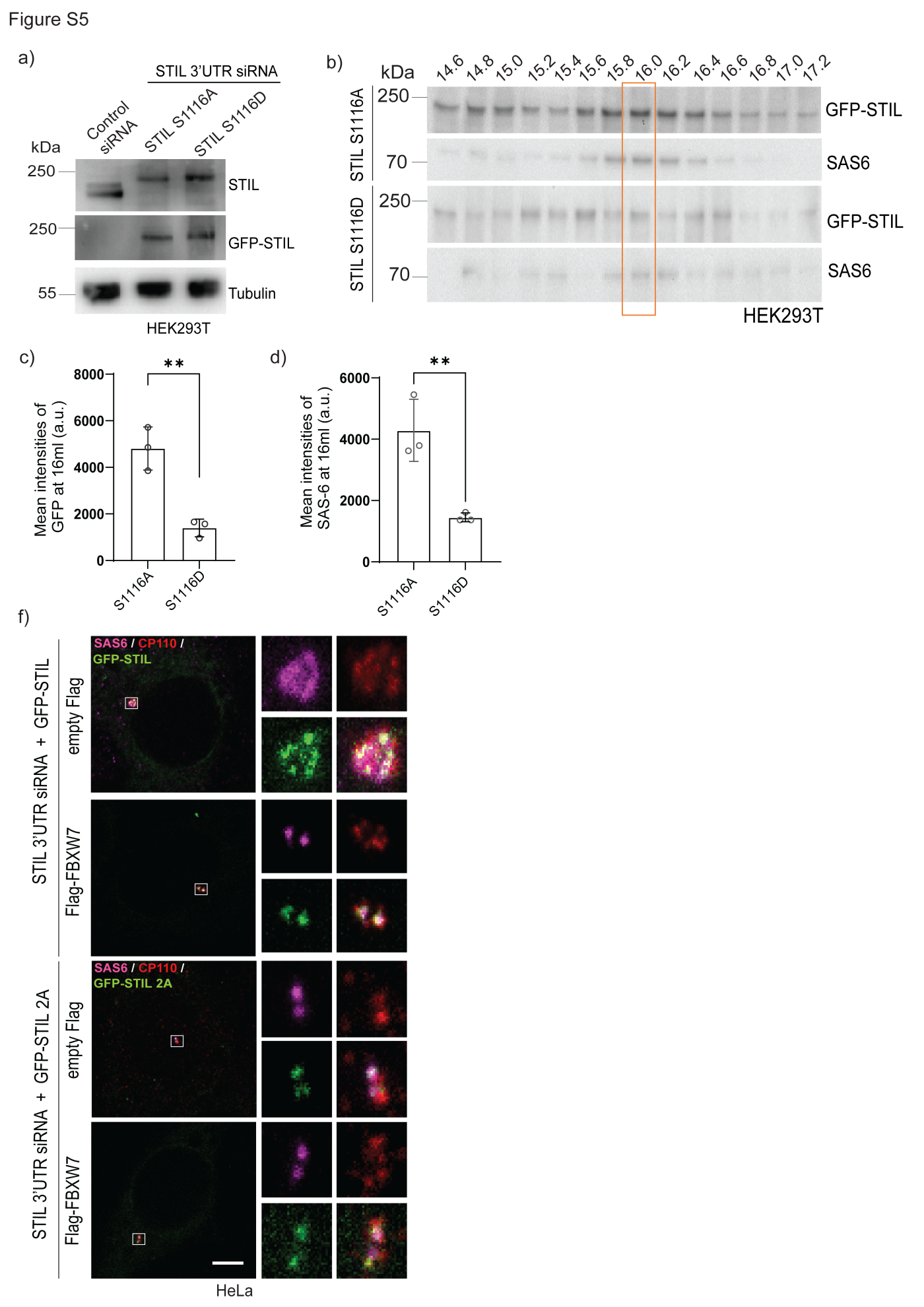


**Figure S5.**  Phosphorylation of Plk4-targeted site in STIL STAN domain is critical for FBXW7-mediated STIL degradation.

a. Lysates of G1/S synchronized HEK293T cells over-expressed with GFP-STIL S1116A and GFP-STIL S1116D with simultaneous depletion of endogenous STIL by 3’UTR siRNA were subjected to Western blot. The volume of cell lysates loaded was ~3.5 times lesser than the volume of the lysates loaded in the size exclusion chromatography shown in S5b. b. Lysates of HEK293T cells depleted of endogenous STIL by 3’UTR siRNA and expressed with GFP-STIL S1116A or GFP-STIL S1116D were loaded onto a Superose 6 size exclusion column. Ethanol precipitated sample fractions collected from the size exclusion chromatography subjected to western blot and probed for the GFP-STIL S1116A vs. S1116D mutant and SAS6. Immunoblots of the samples eluted in the range from 14.6 ml to 17.2 ml are shown since the GFP-STIL and SAS6 proteins were coeluted at and near 16 ml. c. Quantification plot for mean intensities of GFP-STIL S1116A vs. D mutant protein eluted at 16 ml, normalized with tubulin in the GFP-STIL S1116A- and GFP-STIL S1116D- expressed condition. Data = mean ± S.D. **p=0.0041 (Unpaired parametric t-test). d. Quantification plot for mean intensities of SAS6 eluted at 16 ml, normalized with tubulin in the GFP-STIL S1116A- and GFP-STIL S1116D- expressed condition, Data = mean ± S.D. **p=0.0086 (Unpaired parametric t-test). e. Additional representative confocal images of GFP-STIL or GFP-STIL 2A over-expressed in STIL 3’UTR siRNA-treated G1/S synchronized HeLa cells co-transfected with empty Flag or Flag-FBXW7 (48 h) were stained for SAS6 and CP110 (centriole marker). Scale bar = 5 µm.


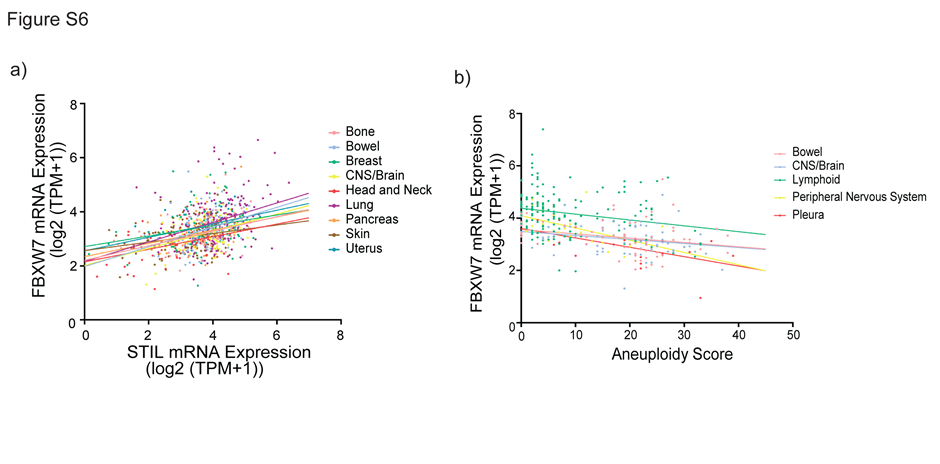


**Figure S6**. Subtype-specific associations between FBXW7, STIL, and aneuploidy.

a. FBXW7 and STIL mRNA expression are positively correlated within individual cancer types. Pearson’s correlations: Bone cancer: r = 0.4192, P = 0.0014; Bowel cancer: r = 0.382, P = 0.0003; Breast cancer: r = 0.2451, P = 0.04; Brain cancer: r = 0.353, P = 0.0003; Head and Neck cancer: r = 0.3719, P = 0.0011; Lung cancer: r = 0.3102, P < 0.0001; Pancreas cancer: r = 0.3014, P = 0.0253; Skin cancer: r = 0.2291, P = 0.0118; Uterus cancer: r = 0.3643, P = 0.0163. b. FBXW7 mRNA expression is negatively correlated with aneuploidy score within specific cancer types. Spearman’s correlations: Bowel cancer: r = -0.31, P = 0.01; Brain cancer: r = -0.2362, P = 0.04; Lymphoid cancer: r = -0.2609, P = 0.001; Peripheral Nervous System cancer: r = -0.6224, P = 0.0001; Pleural cancer: r = -0.4428, P = 0.0506.
